# Microbial hydrogen sinks in the sand-bentonite backfill material for the deep geological disposal of radioactive waste

**DOI:** 10.3389/fmicb.2024.1359677

**Published:** 2024-04-16

**Authors:** Camille Rolland, Niels Burzan, Olivier X. Leupin, Aislinn A. Boylan, Manon Frutschi, Simiao Wang, Nicolas Jacquemin, Rizlan Bernier-Latmani

**Affiliations:** ^1^Environmental Microbiology Laboratory, École Polytechnique Fédérale de Lausanne, Lausanne, Switzerland; ^2^National Cooperative for the Disposal of Radioactive Waste, Wettingen, Switzerland

**Keywords:** bentonite, hydrogen, methanogenesis, sulfate reduction, deep geological disposal, microcosm reactor, opalinus clay

## Abstract

The activity of subsurface microorganisms can be harnessed for engineering projects. For instance, the Swiss radioactive waste repository design can take advantage of indigenous microorganisms to tackle the issue of a hydrogen gas (H_2_) phase pressure build-up. After repository closure, it is expected that anoxic steel corrosion of waste canisters will lead to an H_2_ accumulation. This occurrence should be avoided to preclude damage to the structural integrity of the host rock. In the Swiss design, the repository access galleries will be back-filled, and the choice of this material provides an opportunity to select conditions for the microbially-mediated removal of excess gas. Here, we investigate the microbial sinks for H_2_. Four reactors containing an 80/20 (w/w) mixture of quartz sand and Wyoming bentonite were supplied with natural sulfate-rich Opalinus Clay rock porewater and with pure H_2_ gas for up to 108 days. Within 14 days, a decrease in the sulfate concentration was observed, indicating the activity of the sulfate-reducing bacteria detected in the reactor, e.g., from *Desulfocurvibacter* genus. Additionally, starting at day 28, methane was detected in the gas phase, suggesting the activity of methanogens present in the solid phase, such as the *Methanosarcina* genus. This work evidences the development, under *in-situ* relevant conditions, of a backfill microbiome capable of consuming H_2_ and demonstrates its potential to contribute positively to the long-term safety of a radioactive waste repository.

## 1 Introduction

Confinement of radioactive waste must ensure that humans and environment are protected from radio toxicity. In case of high-level waste confinement period must last for several hundred thousand of years (Hedin, 1997). Switzerland will construct a deep geological repository (DGR) in Opalinus Clay at about 850 m below ground level (Nördlich Lägern). The planned repository consists of a series of blind-end tunnels (the disposal rooms), connected via access galleries. The safe confinement of the stainless-steel canisters containing the waste is ensured by the host rock (the clay rock) and engineered barriers (the backfill). Upon closure of the repository and the establishment of low oxygen conditions, anoxic corrosion of low and intermediate level-waste steel containers is expected to produce up to 25 million m^3^ of hydrogen gas (H_2_), this is roughly 50 times the galleries volume (390,000 m^3^ not including disposal waste chambers) (Diomidis et al., 2016; Leupin, 2016). This significant gas production potentially threatens the integrity of the host rock. A proposed strategy to address this issue is the choice of backfill and sealing materials enabling gas transport out of the disposal rooms in the backfilled access galleries, and colonization of this porous space by hydrogenotrophic microorganisms.

Evidence of microbial H_2_ oxidation in Opalinus Clay porewater, in contact with the host rock, has been previously described *in-situ* (Bagnoud et al., 2016a), or in a cultivation experiment (Boylan et al., 2019), as involving primarily sulfate reduction, which results in the production of bisulfide by consumption of H_2_ and sulfate (Eq. 1):


(1) $$4\;H_{2}+{\mathrm{SO}}_{4}^{2-}+H^{+}\to {\mathrm{HS}}^{-}+4\;H_{2}O$$

In this environment, sulfate is provided by the Opalinus Clay porewater, and the dissolution of sulfate-bearing minerals in the backfill material. In addition, inorganic carbon could fuel methanogenesis and homoacetogenesis serving as additional H_2_ sinks. Homoacetogens have never been observed in Opalinus Clay rock and porewater, and methanogens were detected at very low relative abundance (Bagnoud et al., 2016b; Mitzscherling et al., 2023), although their activity was not established *in situ* (Stroes-Gascoyne et al., 2007; Vinsot et al., 2017).

Our work aimed at probing the growth of hydrogenotrophic microorganisms in backfill material under repository-relevant conditions and at estimating the *in-situ* H_2_ consumption rate of the microbiome using sulfate as a proxy. We chose an 80% (w) quartz sand and 20% (w) Wyoming bentonite mixture as the porous backfill because of its consideration as a potential plug and backfill material for the Swiss low-level waste repository (Manca, 2016). To investigate the adequacy of the concept, flow-through reactors filled with sand-bentonite (80/20 (w/w)) received an influx of Opalinus Clay rock porewater and were amended with H_2_ (100%). The results support the establishment of a microbiome consuming H_2_ gas using sulfate from Opalinus Clay porewater and Wyoming bentonite. The hydrogenotrophic microbial community was composed of sulfate-reducing bacteria (SRB) and methanogens.

Together, these findings show that sulfate reduction and methanogenesis are the dominant drivers of H_2_ consumption in the repository backfill material composed of sand and Wyoming bentonite.

## 2 Materials and methods

### 2.1 *In-situ* equipment

#### 2.1.1 Reactors description

All experiments were carried out in the Underground Rock Laboratory (URL) “Mont Terri” (Switzerland). Four identical cylindrical reactors, with inner dimensions of 12 cm in height × 10 cm in diameter (Supplementary Figure S1), were used for the experiment. The porous matrix simulating the potential repository backfill material (sand-bentonite 80/20 (w/w)) was not in direct contact with the stainless-steel cylinder but held in place by an inner Plexiglas cylinder, including a top and a bottom plate, thus minimizing the interaction with stainless-steel surfaces. The matrix was placed between two layers of coarse sand (1.5 cm thick). These layers were introduced to distribute porewater at the top and bottom and favor the uniform advection of porewater through the sand-bentonite matrix. Sulfate-rich Opalinus Clay porewater was supplied by two titanium inlets at the bottom whereas H_2_ was provided via a long titanium tube ending in the middle of the reactor (Supplementary Figure S1).

**Figure 1:**
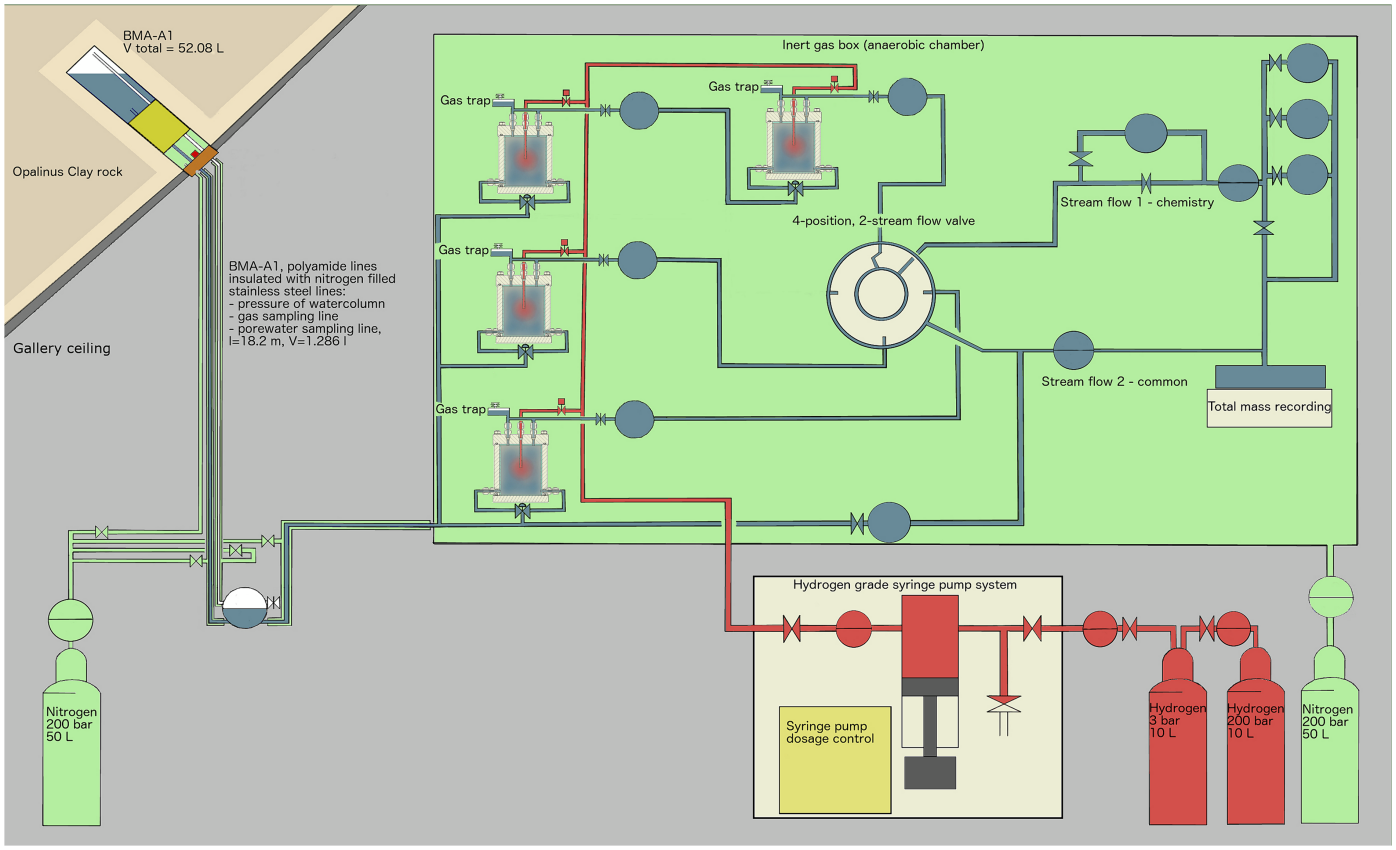
Experimental setup overview: Borehole BMA-A1 (upper left) provides water from the Opalinus Clay formation, which is transported in inert polyamide lines, isolated from the oxic gallery by stainless-steel tubes that are pressurized with 2.5–3.5 bar argon gas. Once within the glovebox, a controlled nitrogen atmosphere (green) is established and the porewater is continuously supplied to four reactors. The outflow is controlled by a pressure controller (PRC) and recorded by flowmeters (FR) and by a scale recording the total mass of porewater outflow. Gas pressure is controlled by pressure controllers (PRC). Once a day, H_2_ (100%) gas was applied remotely using a syringe pump, delivering up to several milliliters of H_2_ to each reactor to stimulate microbial activity. EDZ refers to the Excavation-Damaged Zone: the network of micro and macro fractures created during drilling of the tunnels.

The sand-bentonite mix was produced with kiln-dried quartz sand with a 0.1–0.6 mm grain size. Wyoming bentonite MX-80 was provided by Nagra and is a well-characterized bentonite used in repository research and its mineral composition is given in Supplementary Table S1 (Karnland et al., 2006). Using a 70% ethanol-disinfected porcelain mortar and pestle, all large bentonite aggregates were ground manually to < 0.5 mm. Sand and bentonite were mixed using the four-fold method (Chen et al., 2019). All reactor parts were disinfected shortly before assembly under oxic conditions. No shaking or compacting method was applied. The modules were then transferred to the URL and placed within an anoxic chamber. The experimental setup is presented in Figure 1. Once set in the anoxic chamber and connected to a borehole drilled in Mont Terri URL ceiling, each reactor was saturated with porewater and left to equilibrate in batch mode. The start of the experiment was staggered by one week for each reactor. After 5 to 30 days the water dispensing system was opened and reactors received gravity-driven porewater flow for 73 to 108 days. The borehole (below the packer) and the outer steel tubing surrounding the water lines were flushed with argon to prevent oxygen diffusion into the porewater. H_2_ was supplied to each reactor daily from a computer-controlled H_2_-grade syringe pump (500D Syringe Pump, Teledyne ISCO Inc., Lincoln, Nebraska, United States). The outflow from reactors 2, 3 and 4 were measured by three complementary metal oxide semiconductor (CMOS)-based flow-recorders (SLI-0430, Sensirion AG, Staefa, Switzerland).

Two different set-ups for the reactor experiment were chosen:

Serial setup: Reactors 1 and 2 were connected such that 1 received borehole porewater as its inflow while 2 received the outflow of 1 as its inflow.

Parallel setup: Reactors 3 and 4 were both connected to the borehole water as their inflow.

A sterile sand-bentonite mix, saturated with sterile artificial porewater, and incubated for 30 days under a nitrogen gas phase was used as an abiotic control. More detail is available in the Supplementary material on the reactors (Supplementary Text S1.1), abiotic control (Supplementary Text S1.2), and Opalinus Clay borehole drilling (Supplementary Text S1.3), microbial characterization (Supplementary Text S2.1 and Supplementary Table S2), and chemical characterization (Supplementary Table S3).

### 2.2 Gas phase and porewater monitoring

#### 2.2.1 Sampling

Reactors were sampled weekly for gas and water composition. All valves connecting the reactors to the porewater flow system were closed to avoid any potential backflow. Gas was sampled by attaching a sterile 50 mL serum bottle filled with filtered nitrogen gas (1 bar) to the gas trap and allowing it to equilibrate with the pressure inside the reactor. The bottle was removed when the first drop of water was observed in the bottle. Water was sampled by connecting a sterile syringe equipped with a 0.2 μm filter to the two outflow lines and extracting up to 7 mL. The outflow of gas and water was driven solely by the pressure difference between the interior of the reactor and the glovebox atmosphere. Additionally, borehole porewater was sampled for chemical analysis using the supply line in the glove box.

#### 2.2.2 Gas phase analysis

The gas phase was analyzed qualitatively for the presence of methane using a gas chromatography system (GC-450, Varian, Middelburg, The Netherlands) equipped with a molecular sieve column (CP81071: 1.5 m*1/8″ ultimetal molsieve 13 9 80–100 mesh) and a flame ionization detector.

#### 2.2.3 Porewater analysis

The filtered porewater was aliquoted in the glovebox and conditioned for subsequent chemical analysis of sulfide, ferrous iron, major cations and anions, and trace elements. Sulfide was analyzed using the Cline method (Cline, 1969) and ferrous iron using the ferrozine assay (Stookey, 1970) both with a UV-2501PC spectrometer (Shimadzu, Kyoto, Japan). Major anions and cations were detected and quantified by Ion Chromatography. An IonPac^®^CS13A-5 μm cation-exchange column (Thermo Fisher Scientific Inc., Waltham, Massachusetts, United States), with as gradient eluent 20 mM methane sulfonic acid, was used for major cations, whereas an IonPac^®^ AS18-4 μm anion-exchange column (Thermo Fisher Scientific Inc., Waltham, Massachusetts, United States), with as gradient eluent KOH (from 0.0 to 30 mM), was used for major anions. Trace metals Al, Co, Cr, Cu, Fe, Mn, Mo, Ni, Si, Sr, Zn, were measured using inductively coupled plasma mass spectrometry (ICP-MS) on an Agilent 8,900 Triple Quadrupole (Agilent Technologies Inc., Santa Clara, United States), with all samples prepared in dilutions with 0.1 M HNO_3_ (final concentration, ultra-pure grade, MilliporeSigma, Merck KGaA, Darmstadt, Germany).

### 2.3 Reactor characterization post-retrieval

#### 2.3.1 Reactor disassembly and sampling

**Figure 2:**
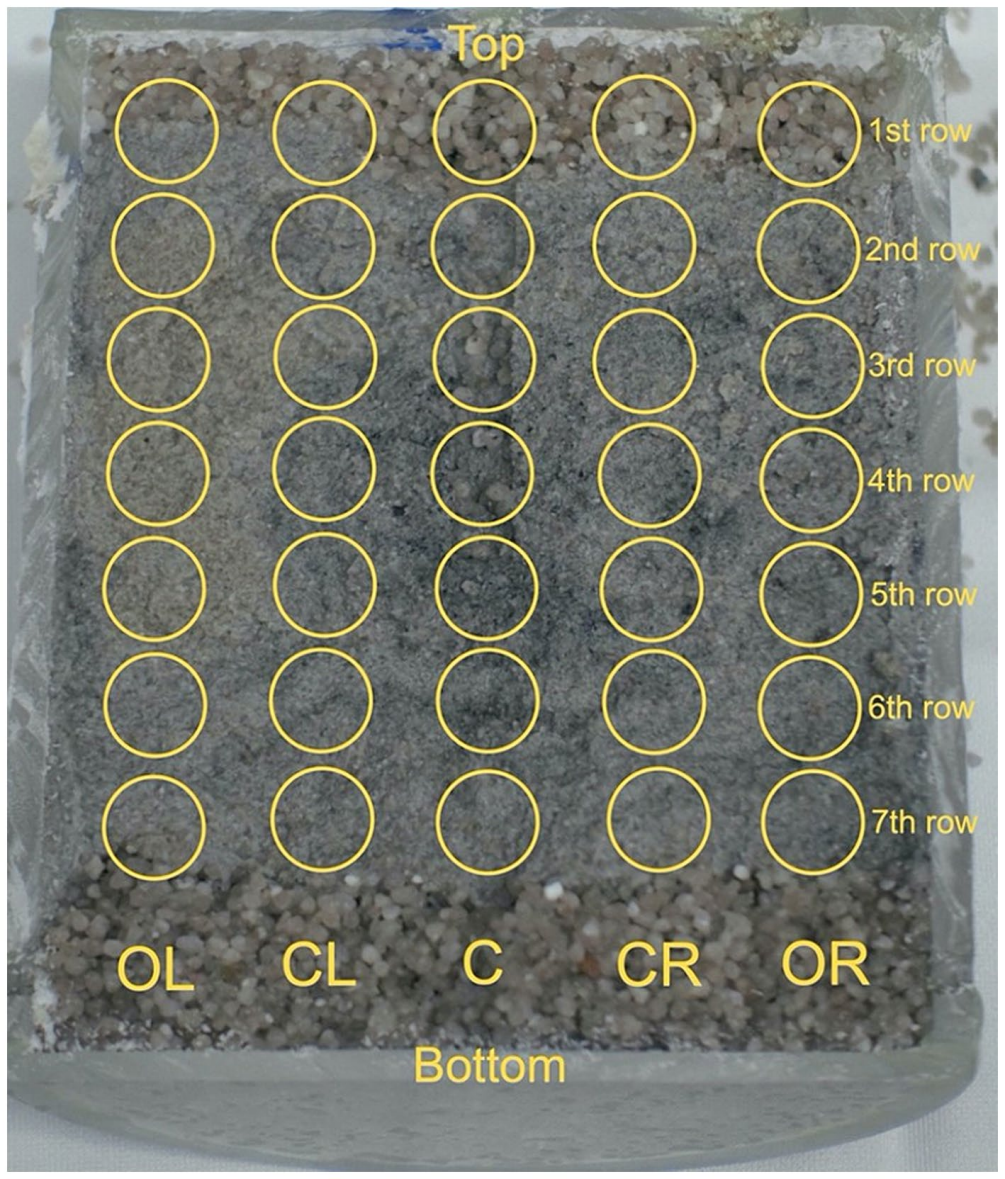
Sand-bentonite sampling grid. Samples were taken at 35 locations from one half of the reactors. Five lines (outer left [OL], center left [CL], center [C], center right [CR] outer right [OR]), representing various radial locations, were sampled at seven heights, numbered from 1st row (top) to 7th row (bottom). Sampling was done under anoxic conditions, using sterile scalpel blades. The collected samples were stored in DNA-free cryo-tubes at −20°C until DNA extraction.

After between 73 to 108 days, the reactors were retrieved from the URL, packed in argon-filled Mylar^®^ bags and transferred to EPFL. The reactors were disassembled within a nitrogen gas-filled MBraun anoxic chamber, and the sand-bentonite core contained within the Plexiglas cylinder was extracted. Sterile, DNA-free (autoclaved for 80 min) cleanroom grade wipes (Spec-Wipe^®^ 7, VWR International LLC, Avantor Inc., Radnor, Pennsylvania, United States) were used to handle the Plexiglas core. A Dremel^®^ 3,000 tool (DREMEL Europe Bosch Power Tools B.V., Breda, Netherlands) was used to cut open the Plexiglas lengthwise. The left half was used for immediate DNA sampling within the glovebox. Five lines (outer left [OL], center left [CL], center [C], center right [CR] outer right [OR]), representing distinct radial locations, were sampled at seven heights, numbered from 1st row (top) to 7th row (bottom), resulting in 35 samples (Figure 2). Equipment was sampled as well (e.g., swab of the steel surface, and organza at the water inlet and outlet). For each sampling spot, a new scalpel blade was used, and the substrate was stored in DNA-free cryotubes at −20°C until DNA extraction. The right half was used for sample collection for subsequent synchrotron-based micro X-ray fluorescence (μXRF) imaging and micro X-ray absorption near edge spectroscopy (μXANES). The remaining part of the left half was embedded in resin for subsequent XRF mapping.

#### 2.3.2 DNA extraction

DNA from the coarse sand and the sand-bentonite were extracted based on an established protocol (Engel et al., 2019a,b), which is a slightly modified protocol of the DNeasy^®^ PowerSoil^®^ Kit (QIAGEN NV, Venlo, Netherlands). The sample substrate ( ~ 0.2 g) was transferred to the kit-provided PowerBead^®^ tubes in a sterile laminar flow hood with flame-sterilized spatulas. The mass was recorded and the sample briefly vortexed within the provided PowerBead^®^ solution. After addition of the kit-provided solution C1 (containing SDS and disrupting agent for cell lysis), the samples were briefly vortexed and incubated for 10 min at 70°C. Homogenization was carried out with a Precellys 24 homogenizer (Bertin Technologies SAS, Montigny-le-Bretonneux, France), for 45 s at 6,000 rpm. The remaining steps were carried out as indicated by the kit manufacturer except for the elution of the extracted DNA, 65 μL of the kit-provided elution buffer C6 were applied to the center of the DNA binding membrane of a MB Spin Column. After centrifugation for 1 min at 8,000 g, the DNA was collected in a 2 mL Soreson^™^ Dolphin tube and stored at −20°C until DNA analysis. More details about the porewater DNA extraction method are available in Supplementary Text S2.1.

#### 2.3.3 16S rRNA gene amplification, sequencing, and statistical data analysis

Briefly, all DNA was quantified using the Qubit^®^ ds-DNA HS Assay Kit (Thermo Fisher Scientific Inc., Waltham, Massachusetts, United States). Amplification of the 16S rRNA gene was performed with a modified protocol of the *Quick*-16S^™^ NGS Library Prep Kit (Zymo Research Corp., Irvine, California, United States) with primers for the V3–V4 region of the 16S rRNA gene (covering both Bacteria and Archaea). Additionally, we took advantage of the fluorescence signal to perform a semi-quantitative assessment of the 16S rRNA gene copy number. qPCR provided insight into the relative quantities of 16S rRNA genes, and thus improved the robustness of the DNA sequencing interpretation (more detail available in Supplementary Text S2.2). The 16S rRNA gene-library was sequenced on an Illumina MiSeq platform (Illumina Inc., San Diego, California, United States) at the Lausanne Genomics Technologies Facility (University of Lausanne, Switzerland), applying 10 pM of the 16S rRNA library with a 15% Phi-X spike in a paired-end 300 bp mode. *t*-tests were achieved with Excel Analysis ToolPak, testing the equality of variance with an *F*-test beforehand, to assess statistical significance in spatial distribution of microbial biomass (Supplementary Figure S8), and to compare the growth in-between reactors (Supplementary Figure S9). More detail is available in the Supplementary material.

#### 2.3.4 16S rRNA gene analysis

Qualities of demultiplexed raw sequencing reads were assessed using FastQC, version 0.11.9 (Andrews, 2019). The processing of demultiplexed raw reads was carried out in R (version 4.1.2, November 2021), and as recommended in the dada2 pipeline documentation (version 1.2). Reads were first filtered and trimmed using the filterAndTrim function. The filtering and trimming parameters were established using Figaro, dockerized version 1.1.2 (Weinstein et al., 2019) with the forward and reverse primer length set to 16 and 24 bp, respectively, and the amplicon length set to 420 bp. With the highest expectation of read retention the following parameters were retained from Figaro output: truncLen = c(289, 191), maxN = 0, maxEE = c(2,1), truncQ = 2, and rm.phix = TRUE. Error rates were then learned using the learnErrors function to perform sample inference with the filtered and trimmed reads. The paired reads were then merged using mergePairs function. Chimeras were removed using the removeBimeraDenovo function with the method set to “consensus.” Finally, the taxonomic assignment of Amplicon Sequence Variants (ASVs) was performed using SINTAX (Edgar, 2016) with the curated Ribosomal Database Project (RDP), trainset 18, release 11.5.

The high-quality reads were imported to ampvis2 (Andersen et al., 2018), which was used to analyze and visualize the sequence data of the sand-bentonite and equipment samples, using heatmaps (genus level), and Principal Component Analysis (PCA, using Hellinger transformation). Unknown taxonomic affiliations are indicated up to the taxonomic level identified. For bubble plots, and relative abundance calculation, the number of reads in the kitome for each Amplicon Sequence Variant (ASV) was removed from the samples, and the result was normalized to the total number of reads in the sample. Spatial maps of selected top ASVs and boxplots of 16S rRNA gene semi-quantification were generated using OriginPro, version 2022b (OriginLab Corporation, Northampton, Massachusetts, United States).

### 2.4 XRF and statistical data analysis

The core was dried in vacuum for 24 h within a MBraun antechamber and embedded using EPO-TEK 301-2 resin (JP Kummer Semiconductor Technology GmbH, Augsburg, Germany). 10 cycles of soft vacuum and ambient pressure were applied. After a low temperature curing for 48 h, a longitudinal cut was performed, approximately 1.5 cm below the plane used for DNA sampling, using a diamond wire saw (MURG 394, WELL Diamond Wire Saws SA, Le Locle, Switzerland). XRF maps of sodium, silicon, phosphorus, potassium, sulfur, calcium, titanium, vanadium, chromium, manganese, iron and nickel (all K-edge) were obtained at the Crystal Growth Facility (EPFL), with an EDAX Orbis PCMicro EDXRF analyzer system (AMETEK Inc., Berwyn, Pennsylvania, United States). Boxplots of counts of iron, sulfur, and calcium fluorescence signals (representing atom-percent values for each element, at scanning spots where the sulfur signal was above 10%) were generated using OriginPro, version 2022b (OriginLab Corporation, Northampton, Massachusetts, United States). *t*-tests were achieved (Excel Analysis ToolPak, previously testing equality of variance with *F*-test) to assess statistical significance of the minerals shifts between biotic reaction and abiotic control (Supplementary Figure S5). More details are available in Supplementary Text S2.3.

### 2.5 Synchrotron μXRF and Fe/S K-edge μXANES

Sand-bentonite samples of about 2.5 cm in length and 1.5 cm in width were obtained from the right half of each reactor in anoxic conditions. The samples were chosen based on visually observable features such as dark color and/or red/brownish halos (Supplementary Figure S2). These samples were placed into custom-built sample holders made of poly-(methyl methacrylate), dried in a high vacuum for 12 h, and stabilized in EPO-TEK 301-2 resin. A minimum of 6 soft-vacuum/ambient pressure cycles were applied to remove trapped gas bubbles. After 48 h of a low-temperature curing, the embedded sample surfaces were gradually exposed by rough removal of excess resin using a Dremel^®^ 3,000 tool. Fine wet grinding using silicon carbide grinding paper of three grit grades (Struers Inc., Cleveland, Ohio, United States) and ethanol, analytical reagent grade (Thermo Fisher Scientific Inc., Waltham, Massachusetts, United States) was performed manually until an even and shiny sample plane was achieved. The prepared samples were sealed within a nitrogen atmosphere by packing in three layers of Mylar^®^. Synchrotron radiation μXRF maps and Fe K-edge XANES spectra were collected at beamline 2–3 of the Stanford Synchrotron Radiation Lightsource at the Stanford Linear Accelerator Laboratory (Menlo Park, California, United States). Fe-XANES data were collected in the energy range of 6,927 eV to 7,520 eV. μXRF imaging and S K-edge XANES was performed at beamline I-18 at the Diamond Light Source (Didcot, Oxfordshire, United Kingdom) using the Data Analysis WorkbeNch (DAWN) (Basham et al., 2015). The S K-edge XANES were collected in the energy range of 2,400 eV to 2,600 eV. Both Fe and S XANES spectra were analyzed using Linear Combination Fitting (Athena software) (Ravel and Newville, 2005).

## 3 Results

### 3.1 Water and H_2_ availability

The experimental set-up aimed to establish a link between the evolving microbiome and the availability of H_2_, the main electron donor, and sulfate, the major electron acceptor. Close to the H_2_ inlet, the availability of H_2_ is high and bioavailable sulfate is expected to be rapidly depleted due to the activity of SRB. As H_2_ was applied daily, the total quantity provided to each reactor is proportional to the duration of the experiment (Table 1). Water flow was controlled by the sand-bentonite back-pressure, resulting in variable flow across time and reactors (Supplementary Figure S3). Reactors 1 and 2 received between 0 and 4 μL/min of porewater (Supplementary Figure S3C); in total, reactor 1 received 10.1 mmoles of sulfate from the porewater while reactor 2 received 8.1 mmoles (Table 1). Reactor 3 received a varying flow of water, on average 4 μL/min until day 57, which sharply increased after day 57 with an average of 20 μL/min until the end of the experiment (Supplementary Figure S3A); in total, reactor 3 received 16.1 mmoles of sulfate from the porewater (Table 1). Reactor 4 received on average 12 μL/min, with a sharp decrease towards the end of the experiment down to 0 μL/min on day 70 (Supplementary Figure S3B); in total, reactor 4 received 22.9 mmoles of sulfate from the porewater (Table 1).

**Table 1:** Sulfate and H_2_ supplied to each reactor, and H_2_ to sulfate molar ratio.

Reactor	Setup	Experiment duration [days]	Sulfate from water [mmol]	Total H_2_ [mmol]	Molar H_2_ to sulfate ratio (water)	Molar H_2_ to sulfate ratio (total)
1	Serial	108	10.1	49.1	4.9	2.7
2			8.1	49.0	6.1	3.0
3	Parallel	73	16.1	32.6	2.0	1.3
4		79	22.9	33.1	1.4	1.1

The molar H_2_ to sulfate ratio (water) accounts only for sulfate from the borehole water, the molar H_2_ to sulfate ratio (total) accounts for the complete dissolution of gypsum from bentonite, providing 8.2 mmol (calculation detailed in Supplementary Text S3).

### 3.2 Sulfate reduction rate

**Figure 3:**
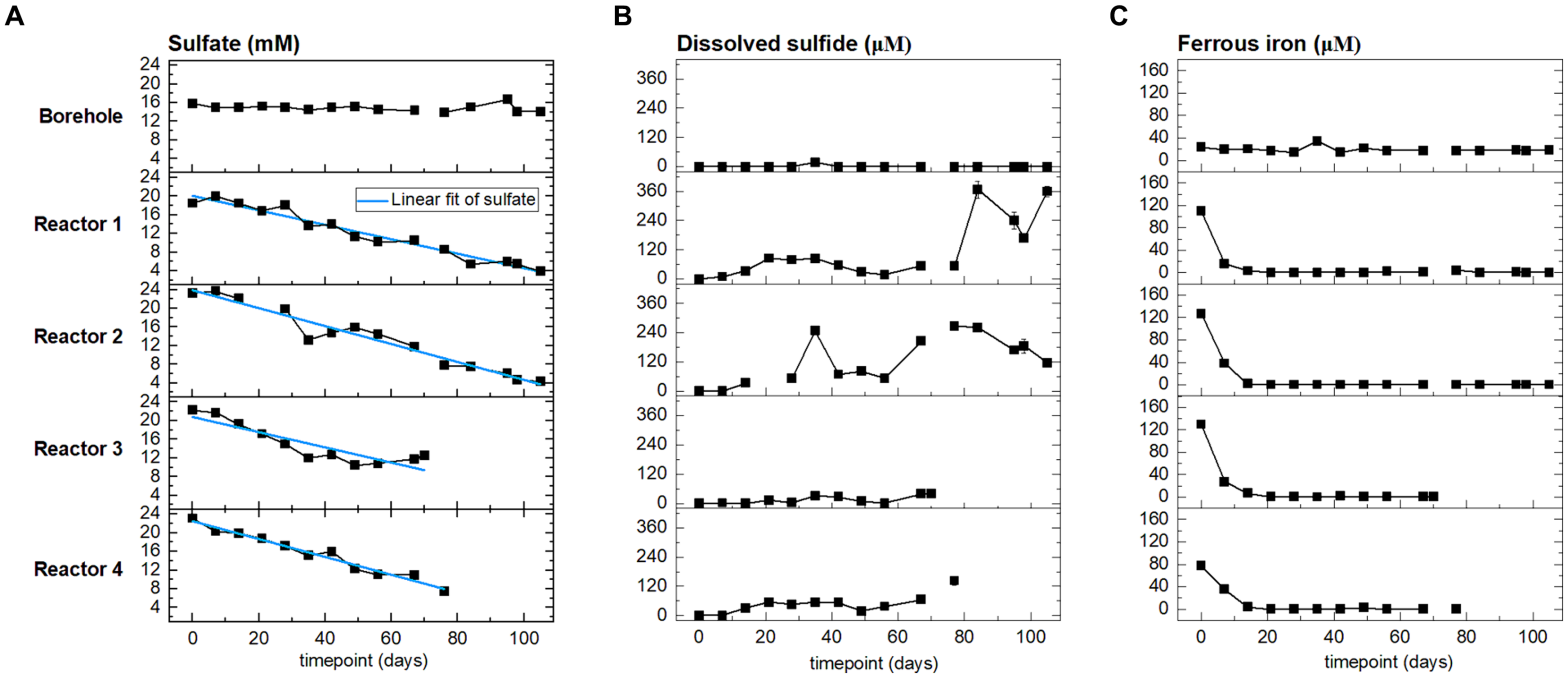
From left to right, time evolution of: **(A)** sulfate, **(B)** sulfide, and **(C)** ferrous iron concentrations at the outlet of the reactors (four bottom rows), and in the borehole porewater (top row). Points are the average of three sampling replicates, error bars represent one standard deviation.

Sulfate is expected to serve as an electron acceptor. In the reactors, sulfate comes from the borehole porewater (approx. 15 mM, Figure 3 and Supplementary Table S3) and dissolution of gypsum from bentonite (Maanoja et al., 2020). Porewater was sampled and analyzed weekly during the H_2_ injection phase, and the evolution of sulfate, sulfide, and ferrous iron is presented in Figure 3. Sulfate concentration followed a decreasing trend from the start of the experiment until the sacrifice of the reactors. Sulfate is reduced to sulfide, which abiotically precipitates with dissolved ferrous iron to form iron sulfide. The initial ferrous iron concentration in reactor 4 (80 μM) was lower than in the three other reactors (120 μM). Ferrous iron was depleted in the four reactors starting on day 10. During the entire experiment, any additional iron coming from the fresh porewater inflow was removed before reaching the outlet. Sulfide was not detected in the borehole porewater. In the reactors, the sulfide concentration remained one order of magnitude lower than the sulfate concentration. In reactors 3 and 4, the concentration of sulfide increased slightly to reach, respectively, 40 μM and 140 μM when sacrificed. In reactors 1 and 2, the sulfide concentration increase was greater, reaching, respectively, 360 μM and 240 μM. At the end of the experiment, the sulfide concentration has increased relative to the initial time point in all four reactors, but it showed temporal variations: a decrease from day 80 to 100 in reactor 1, and from day 80 until the end of the experiment in reactor 2.

Considering sulfate reduction as stoichiometrically linked to H_2_ oxidation, we aimed at calculating the overall sulfate reduction rates in the four reactors during the whole experiment. To this end, we compared two calculations methods: a linear regression and a global mass balance. The sulfate rates calculated are summarized in Table 2. The linear regression model was applied to sulfate concentration curves (linear trend indicated in Figure 3A, detail in Supplementary Table S4). R-squared values obtained for reactors 1, 2, and 4 are above 0.95, which indicates that a zero-order model adequately captures the microbial activity in the reactors.

**Table 2:** Sulfate consumption rate calculated through: (A) Linear regression (Figure 3) and (B) Mass balance (Eq. 2).

	(A) Linear regression			(B) Mass balance
	Rate $\left[\frac{\mu \mathrm{mol}}{\mathrm{day}\;{\mathrm{cm}}^{3}}\right]$	Standard deviation $\left[\frac{\mu \mathrm{mol}}{\mathrm{day}\;{\mathrm{cm}}^{3}}\right]$	Adjusted R-squared	Rate $\left[\frac{\mu \mathrm{mol}}{\mathrm{day}\;{\mathrm{cm}}^{3}}\right]$
Reactor 1	1.548	0.090	0.958	0.35
Reactor 2	1.920	0.124	0.952	0.27
Reactor 3	1.630	0.277	0.794	0.62
Reactor 4	1.911	0.114	0.969	0.52
Average	1.752 ± 0.191			0.44 ± 0.14

For (A), the standard deviation and R-squared values are calculated wit the Linear Regression analysis tool or OriginPro (2022b).

The above sulfate reduction rate was calculated assuming a batch system with the initial sulfate concentration corresponding to that after saturation with borehole porewater during 5 to 30 days (time 0 in Figure 3). This is clearly an assumption that results in an underestimated rate as it does not account for additional sulfate sources: (i) further dissolution of gypsum (dissolution during the initial equilibration released 1.71 to 2.96 mmol, while total gypsum available represents approximatively 8.2 mmol, Supplementary Table S1 and Supplementary Text S3); and (ii) inflow of fresh borehole water with a sulfate concentration of 15 mM. For reactors 1 and 2, the sulfate concentration increases during the 1^st^ week (Figure 3) evidencing the limitations of the batch reasoning.


(2) $${\mathrm{SO}}_{4}^{2-}{\;}_{\mathrm{rate}}=\frac{V_{{\mathrm{in}}_{\mathrm{tot}}}•{\left[{\mathrm{SO}}_{4}^{2-}\right]}_{\mathrm{in}}+n_{\mathrm{gypsum dissolution}\;-}{\sum_{\mathrm{week V}}}_{w}•{\left[{\mathrm{SO}}_{4}^{2-}\right]}_{{\mathrm{out}}_{w}}-\;V_{\mathrm{voids}}•{\left[{\mathrm{SO}}_{4}^{2-}\right]}_{{\mathrm{out}}_{\mathrm{end}}}\;}{\mathrm{days}•V_{\mathrm{voids}}}$$

$V_{{\mathrm{in}}_{\mathrm{tot}}}$ : total water volume injected in the reactor (L) ${\left[{\mathrm{SO}}_{4}^{2-}\right]}_{\mathrm{in}}$ : sulfate concentration in the inflow (mM)n_gypsum dissolution_: quantity of sulfate present in gypsum (10 mmol, Supplementary Text S3)V_w_ and $\;{\left[{\mathrm{SO}}_{4}^{2-}\right]}_{{\mathrm{out}}_{w}}$ total outflow (L) and average concentration (mM) in the outflow during week wV_voids_: volume of voids in the reactor (L) ${\left[{\mathrm{SO}}_{4}^{2-}\right]}_{{\mathrm{out}}_{\mathrm{end}}}$ : sulfate concentration in the outflow at the end of the experiment (mM)

A global sulfate mass balance is considered as a second approximation of the sulfate reduction rate (Eq. 2) and the results summarized in Table 2. The rates are 2 to 5 times lower than the linear reduction rate, and there is a higher variability amongst the reactors.

### 3.3 Sulfur conversion from gypsum to iron sulfide

Due to microbial sulfate consumption, the reactor water is expected to become undersaturated with respect to gypsum, resulting in its dissolution. Furthermore, sulfide produced precipitates with ferrous iron to form iron–sulfur minerals (e.g., mackinawite FeS, greigite Fe_3_S_4_, or pyrite FeS_2_) (Duverger et al., 2020). Patches of black color of the sand-bentonite matrix were observed after incubation with H_2_ (Supplementary Figure S2).

We used XRF for elemental mapping of iron, sulfur, and calcium on one half of each reactor. Sulfur hotspots (i.e., with atomic percent above 10) expected to correspond to pre-existing gypsum (calcium sulfate) or to iron sulfide precipitation were selected to study the correlation between atomic percent of sulfur and calcium, or sulfur and iron. The mean iron to calcium ratio observed at sulfur spots for each reactor significantly shifted from approximatively 1.1 (abiotic control) to 1.5 (reactor 2, *p* < 0.05), up to 4.6 (reactor 1, *p* < 0.05) (Supplementary Figure S5C). Moreover, the decrease in calcium atomic percent at sulfur spots is statistically significant for the four biotic reactors compared to the abiotic control (*p* < 0.00001 for reactor 1, 2, and 4, *p* < 0.005 for reactor 3, Supplementary Figure S5A). In contrast, XRF data do not provide conclusive evidence of the change in iron concentration at sulfur hotspots (Supplementary Figure S5B). In reactors 1, 3, 4 and in the abiotic control (reactor not exposed to H_2_), the average iron to sulfur ratio is 0.45, close to 0.5, corresponding to pyrite, which is initially present in Wyoming bentonite MX-80 (0.60% by weight, Supplementary Table S1). In reactor 2, the iron to sulfur ratio is lower than in the abiotic control (0.17 compared to 0.45, *p* < 0.05). The number of sulfur hotspots in this reactor was greater than in any other reactors, including the abiotic control (225 compared to values ranging from 18 (reactor 4) to 40 (reactor 3)). *t*-test results are detailed in Supplementary Table S5.

We used μXANES to analyze sulfur speciation. For the abiotic control (no H_2_), the best fit for the sulfur data was obtained with gypsum (Supplementary Table S7). For 14 out of the 18 samples exposed to H_2_, the best fits were obtained with pyrite as the main sulfur phase and with mackinawite as the second most abundant phase. In one sample, mackinawite was identified as the main phase by fitting. Poor quality of the standard data impeded obtaining very good values for statistical indicators of the goodness-of-fit, but the values confirm qualitatively adequate fits. Iron XANES spectra analysis confirmed the presence of pyrite and mackinawite in the samples, with higher fit quality than the sulfur XANES spectra (Supplementary Table S8). XANES mapping of iron evidences the presence of a pyrite precipitate of 1 mm^2^ in reactor 2 (Supplementary Figure S6). Unfortunately, the standards did not allow the identification of all the iron phases, with for instance the presence of an unknown iron phase in the middle of a ferrihydrite spot in reactor 3 (Supplementary Figure S7, sample 20 in Supplementary Table S8).

### 3.4 Hydrogenotrophic microbiome dominated by sulfate reducers and methanogens

The 35 sand-bentonite samples collected from the middle plane of each reactor and swabs from equipment parts of the reactors were analyzed to quantify biomass (16S rRNA gene copies number per gram of sand-bentonite) and phylogeny (16S rRNA gene amplicon sequencing). Here, we first discuss results obtained by averaging the relative abundance and biomass quantification results of the samples for each reactor (Figure 2 and Supplementary Tables S9–S11).

When comparing 16S rRNA gene copies number from the initial dry sand and dry bentonite, to the sand-bentonite samples post-incubation, it is clear that growth has taken place within all four reactors: the average 16S rRNA gene copies number per gram of material in reactors post incubation, dry bentonite, and dry sand are respectively: 8.2 × 10^8^, 8.2 × 10^5^, and 3.7 × 10^5^ 16S rRNA gene copies/gram substrate (Supplementary Figures S8, S9). The reactors were run for different durations and the flow rate was variable, resulting in different amounts of total sulfate available (Section 3.1). We expected reactor 4, that received the most water and sulfate (Table 1) to host the highest biomass, but on average, reactor 3 has significantly higher biomass growth than the other reactors (*p* < 0.01 for reactor 1, *p* < 0.05 for reactor 2, and *p* < 0.001 for reactor 4, Supplementary Figure S9 and Supplementary Table S12). The flow in reactor 4 decreased sharply at the end of the experiment [from 20 μL/min at day 60 to 0 μL/min at day 70, with on average 3 μL/min during the last 10 days (Supplementary Figure S3)], meanwhile in reactor 3 it increased up to 32 μL/min, with on average 20 μL/min during the last 10 days (Supplementary Figure S3). Altogether, this suggests that biomass growth measured at the end of the experiment depends on the flow rate (and thus sulfate availability) in the reactors at the end of the experiment rather than during the whole experimental run.

**Figure 4:**
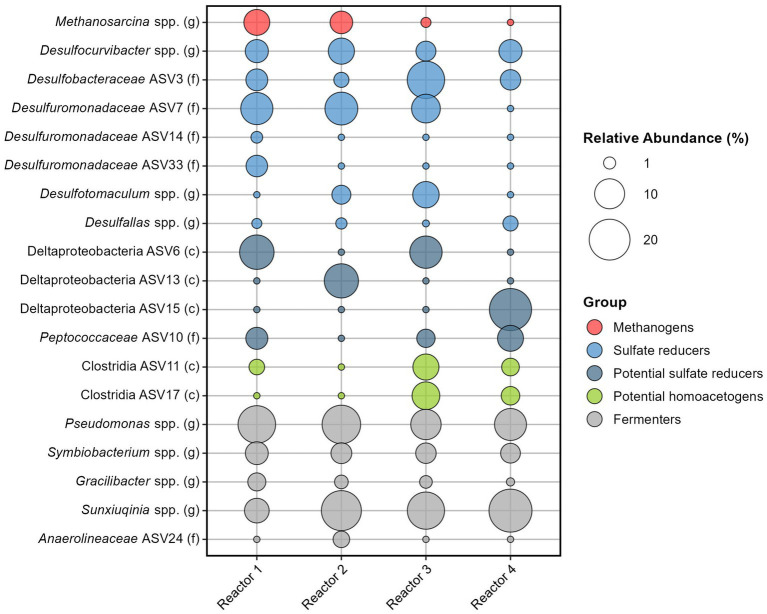
Relative abundance of the 19 most abundant ASVs among the four reactors. Number of reads were averaged for each reactor, and contributions from the Kitome were removed before calculating the relative abundance.

The average relative abundance of the 35 sand-bentonite samples varies across reactors but all include sulfate reducers, methanogens, both known hydrogenotrophs, as well as fermenters (Figure 4 and Supplementary Tables S9, S10).

Among sulfate reducers, *Desulfocurvibacter* genus has a similar average relative abundance in the four reactors, ranging from 3.8% (reactor 3) to 7.3% (reactor 2). Sulfate reducers from the Proteobacteria phylum are well represented with four ASVs identified to be part of the *Desulfobacteraceae* or *Desulforomonadaceae* families (Figure 4). *Desulfuromonadaceae* (ASV7) has the highest overall relative abundance (6.8%, Supplementary Tables S9, S10). Other sulfate reducers are Firmicutes, with *Desulfotomaculum* spp. identified in reactors 2 and 3 and *Desulfallas* spp. identified in all reactors. Three ASVs from the Deltaproteobacteria class could not be assigned down to the genus level, however, many families of this class are sulfate reducers. Data analysis using the RDPnaive algorithm (which is less restrictive) predicted the attribution of ASV6 to the order Desulfobacterales and of ASV10 from the family *Peptococcaceae* to genus *Desulfallas* (data not shown). If ASVs 6 and 10 are indeed sulfate reducers, the relative abundance of sulfate reducers is high in all reactors: 38.7% (reactor 2), 42.2% (reactor 4), 46.9% (reactor 1), and 50.2% (reactor 3) (Supplementary Tables S9, S10). Taking ASVs individually, we observe that only in reactor 3 does an ASV corresponding to a sulfate reducer have the highest relative abundance on average (ASV3 from *Desulfobacteraceae* family with 16.5%). In the other reactors, the most abundant ASV is from the genus *Pseudomonas* or *Sunxiuqinia*.

*Methanosarcina* genus is the only representative of methanogens. Averaging the 35 sand-bentonite samples in each reactor, the genus was detected as the 4^th^, the 7^th^, and the 13^th^ most abundant ASV in reactors 1, 2, and 3, respectively. This genus was also detected in reactor 4, but the average number of reads was lower than the number of reads in the Kitome, and thus the presence of this genus in the sand-bentonite matrix could not be confirmed. Activity of this methanogen was confirmed via the detection of methane in all four reactors during the experiment (Supplementary Figure S4). Indeed, detection of methane in the gas phase of reactor 4 supports the presence of *Methanosarcina* spp. in this reactor, despite its low abundance based on reads count.

Fermenters do not participate in H_2_ oxidation but foster availability of low molecular weight organic carbon substrates for the growth of H_2_ oxidizers. Attribution of fermentative metabolism based on genus is an approximate endeavor as many bacteria capable of fermentation are also able to catalyze other reactions. For instance, members of the genus *Pseudomonas* can ferment many substrates but also carry out denitrification (Carlson and Ingraham, 1983) or iron reduction (Vasil, 2007). Therefore, while we classify *Pseudomonas* and *Sunxiuqinia* genera as fermenters here, they could also be involved in other metabolisms. These two genera are part of the top five most abundant ASVs, with relative abundance ranging from 10.3 to 17.6%, and 6.4 to 22.4%, respectively. Other putative fermenters are from *Symbiobacterium* genus., with a homogeneous relative abundance across the reactors, and from *Gracilibacter* genus.

**Figure 5:**
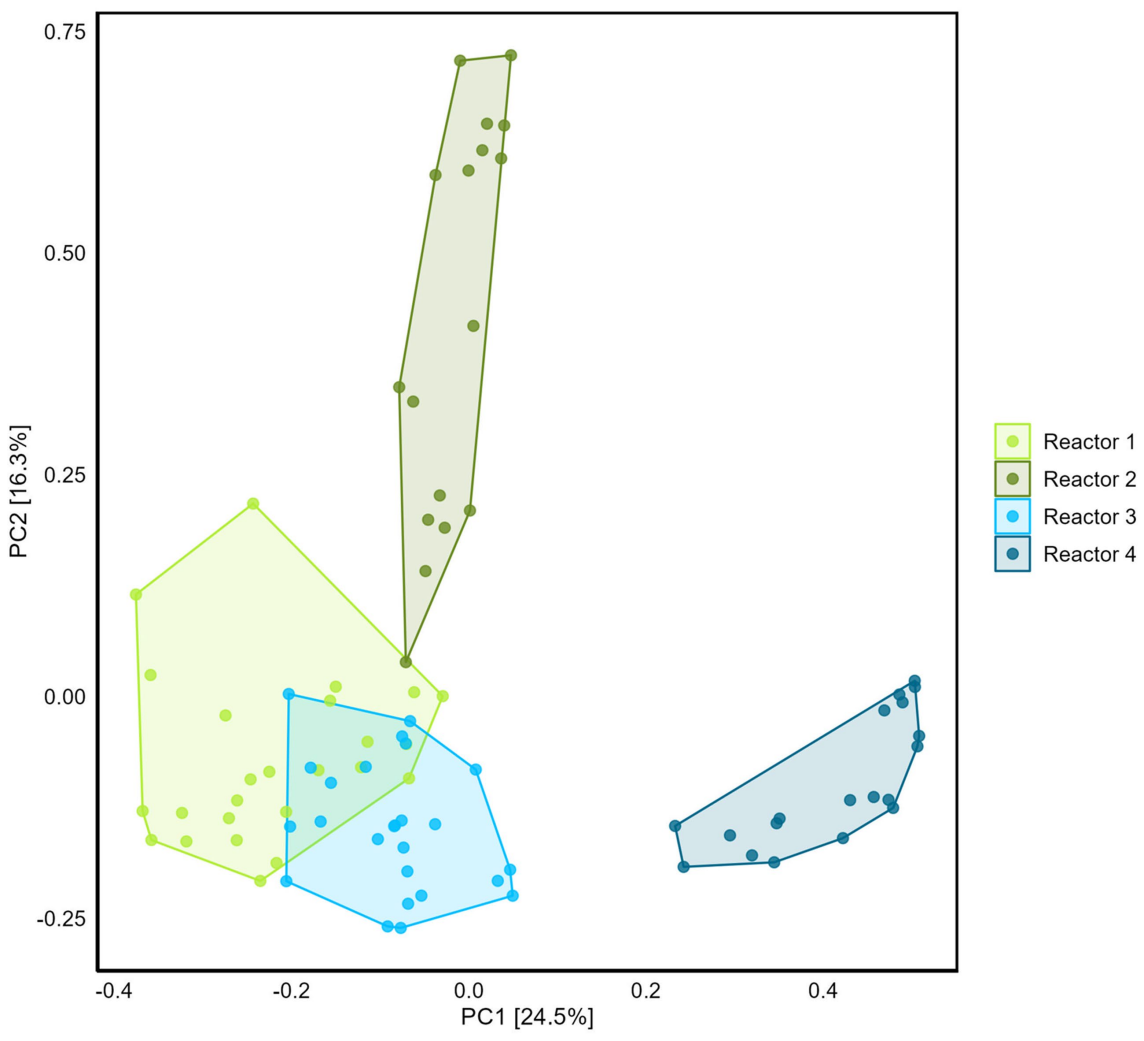
Comparison of the four reactors microbiome using Principal Component Analysis (with Hellinger transformation). Percentage of the variance corresponding to the principal components (PC) is indicated in the brackets for each axis.

Differences between reactor microbiomes were evidenced using PCA (Figure 5). Reactors 1, 2, and 3 present similarities with the overlapping of the clouds formed by the samples plotted on the two first principal component axes, while reactor 4 plots separately.

The PCA of the microbial community associated with equipment evidences similarity amongst the four reactors (Supplementary Figure S10). The top ASVs are from the genera *Pseudomonas, Desulfocurvibacter, Desulfobacteraceae (ASV3), Methanosarcina, Gracillibacter, Desulfallas, Sunxiuqinia* (Supplementary Table S13). *Methanosarcina* genus was preferentially located at the water inlet of reactors 1 and 2 (data not shown). *Desulfocurvibacter* genus was observed at the water inlet, outlet (where it has the higher relative abundance), and on the H_2_ tube, suggesting the dependence of this genus on H_2_ (data not shown).

### 3.5 Microbiome spatial distribution

The spatial distribution of biomass and ASVs in each reactor also contains useful information that can be mined as a result of the spatially-resolved sampling. Biomass concentration was compared across vertical columns for each reactor and evidenced that growth had taken place mainly in the middle vertical column in each reactor, where the highest availability of H_2_ is expected (Supplementary Figure S8). Indeed, for all reactors, at least one of the off-center columns showed significantly (*p* < 0.05) lower 16S rRNA gene copies number per gram of substrate than the central column (Supplementary Table S13). Notably, in reactor 3, the number of 16S rRNA copies is lower on either side of the central column (*p* < 0.05). For reactor 2, and reactor 4, three columns out of four have significantly lower number of 16S rRNA copies than the central one (*p* < 0.05). For reactor 1, only the outer left column presents significantly lower number of copies (*p* < 0.05). In this reactor, growth appears to have taken place homogeneously. *t*-test results for all reactors are detailed in Supplementary Table S13.

**Figure 6:**
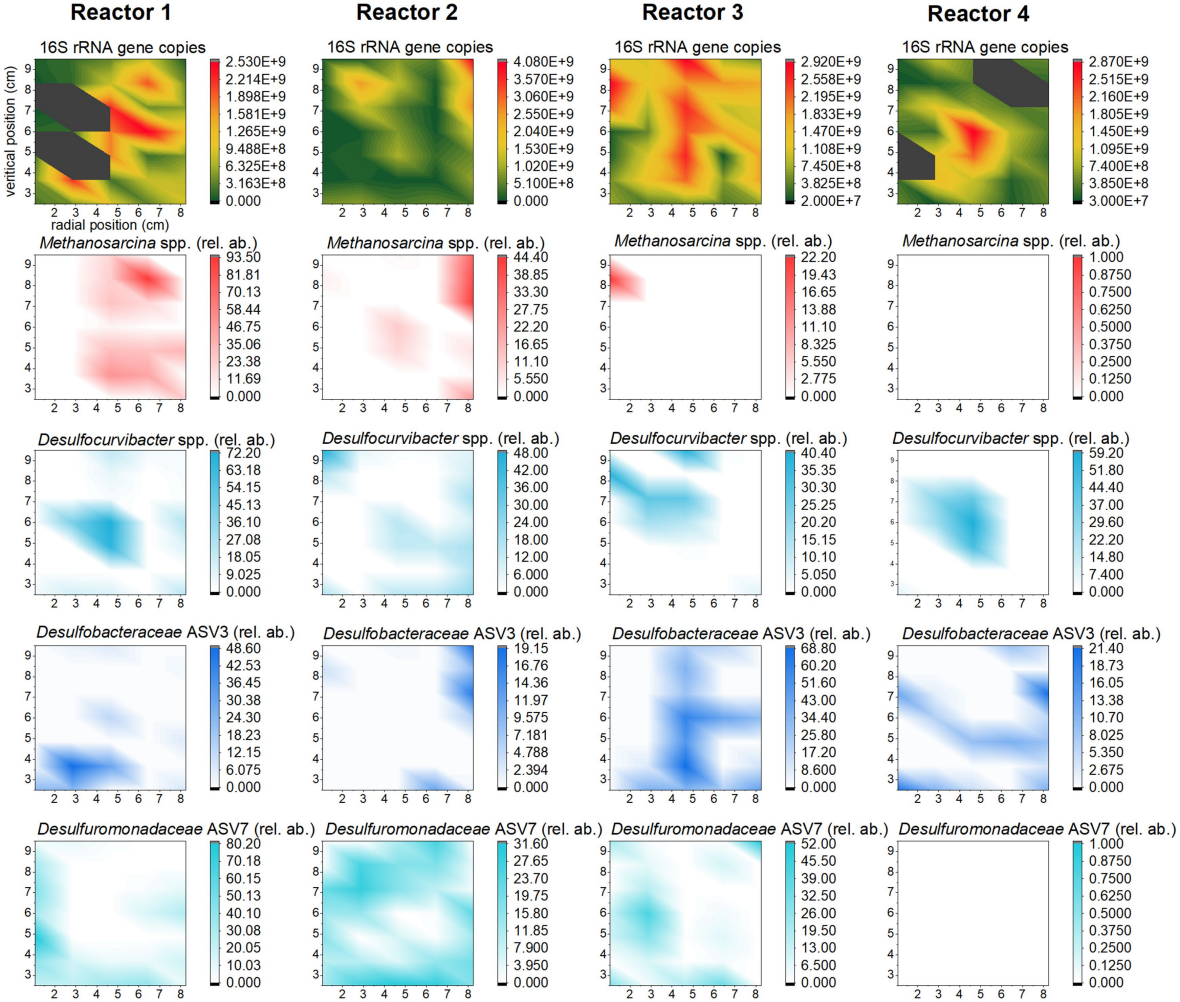
Distribution of biomass (top row) and selected ASVs (other rows) in the four reactors. The heat maps were achieved using OriginPro (2022b), with extrapolation of the data from the 35 sampling spots (Figure 2). The color scale corresponds to the number of 16S rRNA gene copy number per gram of substrate for biomass distribution, and to the distribution of the local abundance of the methanogen and sulfate reducers relative to total biomass, for each sampling spot.

Maps of individual taxa within the microbiome (Figure 6) show that sulfate reducers, particularly member of *Desulfocurvibacter* genus, grow around the H_2_ outlet. In reactor 3, *Desulfocurvibacter* spp. were outcompeted by other SRBs, and the middle column of the reactor is occupied by a member of *Desulfobacteraceae* family (which is the most abundant ASV in this reactor). In reactors 1, and 2, the most abundant sulfate reducer (from the *Desulfuromonadaceae* family) occupies mainly the outer left and bottom of the reactor (reactor 1) or is homogeneously distributed (reactor 2).

In reactor 2, *Methanosarcina* spp. grew near the H_2_ outlet, with a similar growth pattern as that encountered for *Desulfocurvibacter* spp. However, in this reactor the highest relative abundance of *Methanosarcina* spp. is observed at the top right corner and corresponds to the highest number of 16S rRNA gene copies in this reactor. In reactor 1, members of *Methanosarcina* genus grew where there is no SRB growth; in reactor 3, they grew at the top left corner, overlapping with members of *Desulfocurvibacter* genus.

## 4 Discussion

### 4.1 Sulfate reduction and H_2_ oxidation rates

The sulfate reduction rate was calculated using two approaches: a linear regression, and a global mass balance. The rates obtained by linear regression were two to five time higher (on average, $1.752\pm 0.191\frac{\mu \mathrm{mol}}{\mathrm{day}\;{\mathrm{cm}}^{3}}$ ) than the global mass balance calculation (on average, $0.44\pm 0.14\frac{\mu \mathrm{mol}}{\mathrm{day}{\mathrm{cm}}^{3}}$ ). The rates obtained by linear regression are similar for the four reactors (RSD of 11%, highest rate in reactors 2 and 4, and lowest in reactors 1 and 3, but no statistically significant differences), despite different running conditions (duration, water flow, parallel/serial setup). The global mass balance captures differences (RSD of 24%), especially related to water flow (highest rate in reactors in the parallel setup, lowest rate in reactors in the serial setup). The linear regression considered a batch system with no additional input of sulfate and gave an average value for the entire duration of the experiment. On the contrary, the global mass balance takes into consideration sulfate inputs (dissolution, fresh borehole water) and loss of sulfate in the outflow (Eq. 2). The mass balance is thus more complete and is considered to be a better model. The difference between the two rates highlights the limited quantitative information provided by the evolution of the concentration at the outflow, given the existence of preferential flow paths, and the heterogeneous sulfate dissolution and consumption in the porous matrix.

The range of the rates calculated is five to ten-fold higher than the sulfate reduction rate of 0.14 to 0.20 $\frac{\mu \mathrm{mol}}{\mathrm{day}\;{\mathrm{cm}}^{3}}\;$ reported by Bagnoud et al. (2016c) for a similar Opalinus Clay rock derived microbial community but within a porewater-filled borehole. This suggests that the porous material (representing backfill in the future repository) provides more favorable growth and/or metabolic conditions. Considering a 1:4 stochiometric ratio, the H_2_ oxidation rate was estimated to be 2–7 $\frac{\mu \mathrm{mol}}{\mathrm{day}\;{\mathrm{cm}}^{3}}$ using the average of the results from the mass balance and the linear regression, respectively. Literature-reported rates of H_2_ oxidation by sulfate reducers cover a wide range: 1.2 to 312 $\frac{\mu \mathrm{mol}}{\mathrm{day}\;{\mathrm{cm}}^{3}}$ as reported by Thaysen et al. (2020). Dohrmann and Krüger (2023) measured H_2_ oxidation in the formation fluid in a natural gas field and found the pressure-dependent rate to vary between 0.123–0.325 $\frac{\mu \mathrm{mol}}{\mathrm{day}\;{\mathrm{cm}}^{3}}$ at ambient pressure and to increase up to 4 $\frac{\mu \mathrm{mol}}{\mathrm{day}\;{\mathrm{cm}}^{3}}$ at 100 bar.

It is most useful for a DGR to compute the H_2_ consumption rate as a function of the volume of backfill material. The rate is estimated to be 0.9–3.2 $\frac{\mathrm{mol}}{\mathrm{day}\;m_{\mathrm{backfill}}^{3}}$ (calculation detailed in Supplementary material, Supplementary Text S4). Considering a production of 25 million m^3^ (900 × 10^6^ mol) of H_2_ during a safety period of 1,000 years for the storage of radioactive waste, and constant H_2_ oxidation rate, less than 1% of the backfill would be required for the microbial consumption of all the H_2_ produced. There are clearly many approximations associated with these calculations: for instance, partial saturation of the backfill and limited water availability, sulfate depletion, or the dependence of microbial activity on temperature and pressure (Dohrmann and Krüger, 2023). In reality, one can expect variable H_2_ production and consumption rates as a function of time.

In the work by Bagnoud et al. (2016c), the stoichiometry of sulfate reduction was observed to be close to 8.5 moles of H_2_ per mole of sulfate reduced, which was attributed to other reduction reactions coupled with H_2_ oxidation (i.e., reduction of intermediate valent state sulfur species, clay iron reduction) and H_2_ losses. In our study, sequencing of the 16S rRNA gene did not provide conclusive information on the presence of homoacetogens and iron reducers. Nonetheless, given that ASVs from the class Clostridia, and the family *Desulfuromonadaceae* were detected, and are taxa that harbor homoacetogens and iron reducers, respectively, we cannot exclude a role for those metabolisms in H_2_ consumption. Furthermore, the presence of hydrogenotrophic methanogens highlights the limitations of using sulfate reduction as a proxy for H_2_ oxidation rate.

Mapping sulfate-reducing taxa indicates that, while some genera (e.g., *Desulfocurvibacter*) have grown close to the H_2_ tube outlet, and, thus, their growth may be limited by H_2_ availability, other ASVs capable of sulfate reduction were distributed homogeneously within all reactors. The homogeneous distribution suggests the use of other electron donors (e.g., low-molecular weight organic acids). Bentonite can release organic carbon (Maanoja et al., 2020) which could serve as an electron donor. So can the degradation of autotrophic biomass. If the contribution of organic carbon to sulfate reduction is substantial, the sulfate reduction rate could be an overestimation of the H_2_ consumption rate. However, lactate and acetate were never detected in the outflow and total dissolved organic carbon was not measured, preventing meaningful assessment of the contribution of organic carbon to this metabolism. Rates measured by Bagnoud et al. (2016c) presented evidence that sulfate reduction did not account for all H_2_ consumption as the amount of H_2_ consumed was 8.5-fold greater than that calculated by the stoichiometric ratio with sulfate. However, in the same experiment, consumption of organic carbon supports the occurrence of heterotrophic growth, possibly of the sulfate reducers (Bagnoud, 2015). For the present study, this suggests that not all sulfate reduction is attributable to H_2_ oxidation, but nonetheless we can expect that more H_2_ is consumed than sulfate reduced, due to other metabolisms.

Overall, the reported rate of sulfate reduction can be viewed as a conservative range due to three important restrictions. First, the sulfate-reducing biofilm within the reactor was not fully developed as evidenced by the remaining sulfate within the outflow water after more than 100 days of daily H_2_ injections (Figure 3). Second, the H_2_ oxidation rate calculated via sulfate reduction observed at the outflow does not account for sulfate consumption in the unconnected porosity. Third, the H_2_-oxidizing community within the sand-bentonite matrix is not exclusively composed of SRB, but also of methanogens. Thus, the actual H_2_ oxidation rate is likely to be higher.

### 4.2 Establishment of a hydrogenotrophic microbiome

To decipher whether the most abundant ASVs originated from the borehole water or the sand or bentonite solids, DNA from the dry sand and clay, and from borehole porewater, was extracted and sequenced (Supplementary Tables S2, S14). The microbiomes from the borehole porewater and the dry materials are clearly different from that of the reactors, except for *Pseudomonas* genus, that is present everywhere. This ASV is one of the most abundant in the borehole water and was detected in the coarse sand and in two out of three dry bentonite samples as well. None of the other 25 most abundant ASVs from reactors, dry sand, dry bentonite, or borehole water, overlap. The microbiome obtained from dry clay materials may not represent the full diversity of the community due to the inability to detect low-abundance species. This is evidenced when comparing the microbial diversity of dry clay and that of the same clay placed in water with an electron donor/acceptor (Vachon et al., 2021). Therefore, it is not a trivial exercise to pinpoint the origin of the microbial community identified in the 4 reactors. However, similarity in the microbial community amongst three of the four reactors (Figure 5), even though their preparation occurred up to a month apart, suggests that the origin of the microbiome is likely to be the dry materials or the borehole porewater, and not the equipment or preparation and handling conditions. Members of *Gracillibacter*, and *Peptococacceae* genera could originate from the borehole water (number of reads > 500). ASVs from *Paenibacillaceae*, which was among the most abundant families in the borehole water, did not grow in the reactors. ASVs from the order Clostridiales represent some of the most abundant genera in the borehole porewater, but do not correspond to the ASVs from the class Clostridia detected in the reactors. Finally, surprisingly, few reads (88 reads on average, with 45 reads in the Kitome considered to be contamination) were detected in the reactor for the most abundant borehole porewater sulfate reducer, *Desulfosporosinus* genus, suggesting that it did not thrive in the sand-bentonite porous medium amended with H_2_. Altogether, comparing the microbiomes of the dry materials, the borehole porewater, and the reactors did not allow to conclusively disentangle the origin of the most abundant genera in the reactors.

Sampling the internal surfaces of the equipment (water inlet/outlet, gas trap, gas inlet, steel and Plexiglas surfaces) provided the opportunity (i) to decipher the potential contribution of the microbiome associated with the equipment to sulfate consumption and methane production, (ii) to identify whether conditions in the sand-bentonite support growth of a matrix-specific community, distinct from the one on the equipment surfaces. PCA highlighted a marked difference of the community in the sand-bentonite in reactor 4 compared to that in the three other reactors, which was not reflected in the sulfate reduction rate. Contrary to the sand-bentonite matrix, samples obtained from the equipment were similar in the four reactors (overlapping of clouds on equipment PCA, Supplementary Figure S11) and *Desulfocurvibacter*, *Pseudomonas*, and *Methanosarcina* genera were detected with the highest relative abundance (Supplementary Table S13). This observation suggests that the equipment community might contribute non-negligibly to sulfate reduction rates, and would explain why the rates from the linear-regression are similar across reactors despite differences in the sand-bentonite matrix microbial community. Additionally, it suggests the development of distinct communities within the sand-bentonite porous medium in each reactor that differ from that on the equipment. Thus, the sand-bentonite microbial community in each reactor may have been shaped by the specific flow rate, that impacts the availability of solutes as well as the ratio of H_2_ to sulfate (Table 1).

Monitoring the outflow sulfate concentration associated with the linear-regression analysis has the advantage of giving information on temporal variations in sulfate consumption, and thus, the activity of sulfate reducers. The linear-regression fitted the sulfate consumption data for reactors 1, 2, and 4 adequately (R-squared superior to 0.95) indicating a steady-state activity during the experiment, but less so for reactor 3 (R-squared of 0.79). The low R-squared value for the reactor 3 linear model is explained by an increase in sulfate concentration after 57 days, which is likely due to a steep increase in the water inflow rate (Supplementary Figure S3). Sulfate may not have been consumed by the microbial community rapidly enough to overcome this abrupt increase. Another possibility is the creation of preferential water flow paths not yet colonized by sulfate-reducing communities. If only the low water flow regime is considered (first 50 days) for reactor 3, a linear sulfate reduction rate of 2.569 $\frac{\mu \mathrm{mol}}{\mathrm{day}\;{\mathrm{cm}}^{3}}\;$ is observed, with a R-squared of 0.96. This is the highest rate measured in the four reactors, and matches sequencing results which indicated that, on average, SRB were present with a higher relative abundance in this reactor (Results 3.4). However, the underlying reasons for greater growth and activity of SRB in this specific reactor are not understood.

### 4.3 Evidence for H_2_ consumption via methanogenesis

Methane was detected in the gas phase of all reactors. *Methanosarcina* spp. are versatile methanogens capable of all three methanogenic pathways (Madigan et al., 2018), thus no direct conclusion of its role in H_2_ consumption can be extracted from this system. However, its growth and the detection of methane strongly support methanogenesis as an important metabolism stimulated by H_2_ (directly or indirectly). Methanogenesis consumes 5 moles of gas, for 1 mole of CH_4_ produced, thus participating to pressure decrease despite gas production (4H_2_ + CO_2_ ➔ CH_4_ + 2H_2_O). The average relative abundance of *Methanosarcina* spp. was higher in reactors 1 and 2, which received the least water, and thus the least sulfate. Detection of this genus in reactor 3, which received variable water flow rate (very low for the first 2/3 of the experiment and high in the last third), suggests growth during the first period when the reactor received little sulfate. The number of reads associated to *Methanosarcina* spp. was below that of the kitome in all samples from reactor 4, which received by far the highest total porewater volume (Table 1). Not taking into consideration gypsum dissolution as a source of sulfate, total H_2_ to sulfate ratio in reactors 1 and 2 is above the expected stoichiometry (respectively 6.2:1 and 4.9:1 vs. 4:1, Table 1), meaning that the sulfate provided could not have oxidized all H_2_, pointing to CO_2_ as a potential additional electron acceptor. Indeed, there is circumstantial evidence for hydrogenotrophic metabolism by members of *Methanosarcina* genus by the fact that its growth is localized at the point at which H_2_ is delivered into reactor 2. In addition, the relative abundance of *Desulfocurvibacter* genus is lower than in reactors 1 and 4 at the same spot. Therefore, *Methanosarcina* genus is hypothesized to be outcompeted by SRB when sulfate is not limited but grow actively in case of sulfate depletion.

Representatives of Archaea have been detected in Opalinus Clay rock at very low abundances (Stroes-Gascoyne et al., 2011; Mitzscherling et al., 2023), and there is no direct evidence of their activity to date (Poulain, 2006; Stroes-Gascoyne et al., 2007; Vinsot et al., 2017). They are also found at extremely low abundance in borehole water (only the genus *Methanolobus* was detected) (Vinsot et al., 2014; Bagnoud et al., 2016a). This is likely due to the absence of an abundant electron donor and the abundance of sulfate, both of which favor SRB over methanogens. The higher substrate affinity of SRB to H_2_ as compared to that of methanogens and homoacetogens accounts for SRB’s competitive advantage (Kristjansson et al., 1982; Muyzer and Stams, 2008). When methanogens and SRB are present, the latter can maintain H_2_ at a low steady-state concentration, at which methanogenesis is endergonic (Hoehler et al., 2001). In the subsurface, methanogen-, or methanogen/homoacetogen-dominated communities were detected in water with sulfate concentration around 1 mM (i.e., 15-fold lower than in Opalinus Clay porewater) (Kotelnikova and Pedersen, 1997, 1998; Chapelle et al., 2002). In the sand/bentonite backfill, methanogens could be indigenous to bentonite, as evidenced by the detection of methane during bentonite incubation (Maanoja et al., 2020). Unfortunately, phylogeny was not investigated in that work.

This result highlights that metabolisms other than sulfate reduction should be considered as microbial H_2_ sinks in a DGR backfill.

## 5 Conclusion

This work demonstrates the natural occurrence of a hydrogenotrophic microbiome in a backfill-like porous material mimicking the one planned for the deep geological repository of radioactive waste in the Swiss concept. The origin of the microbiome, whether porewater, sand-bentonite or equipment, was not ascertained. The microbiome was dominated by sulfate reducers as sulfate was the main electron acceptor. In addition to the dissolved sulfate in the borehole porewater, the dissolution of gypsum from the backfill material provided substantial sulfate and was promoted by microbial activity. The sulfide formed was scavenged by iron. The rate of sulfate consumption was higher than in previous studies in a Opalinus Clay borehole, indicating that the backfill material provides a propitious environment for microbial growth and activity. The microbiome composition varied with the amount of water provided to the reactors. In the case of a low flow rate, methanogens were detected in the porous medium, and their activity was validated by the detection of methane. Hydrogenotrophy by methanogens was supported by their spatial localization, and increased abundance when fresh porewater was less available, creating zones of local sulfate depletion. This suggests that methanogens can outcompete sulfate reducers when sulfate is depleted.

Using sulfate consumption as a stoichiometric proxy for H_2_ oxidation represents an underestimate of the H_2_ oxidation rate: first, because the amount of sulfate present is underestimated due to gypsum dissolution; and second, because of the presence of other electron acceptors (e.g., ferric iron, inorganic carbon), and microorganism able of carrying out these other metabolisms. Direct quantification of H_2_ oxidation will be required to build predictive models of the evolution of the gas phase in a DGR.

## Data availability statement

The datasets generated and analyzed for this study can be found in the Zenodo repository using the following URL: https://zenodo.org/records/10352904.

## Author contributions

CR: Methodology, Validation, Visualization, Writing – original draft, Writing – review & editing. NB: Conceptualization, Investigation, Methodology, Visualization, Writing – original draft, Writing – review & editing. OL: Conceptualization, Writing – review & editing. AB: Investigation, Writing – review & editing. MF: Investigation, Writing – review & editing. SW: Investigation, Writing – review & editing. NJ: Investigation, Writing – review & editing. RB-L: Conceptualization, Supervision, Writing – original draft, Writing – review & editing.
