# Supplementary material for: Microbial hydrogen sinks in the sand-bentonite backfill material for the deep geological disposal of radioactive waste
Source: Front Microbiol. 2024 Apr 16;15:1359677. doi: 10.3389/fmicb.2024.1359677 (PMC11060177; doi:10.3389/fmicb.2024.1359677)
Supplement: Supplementary file 1 [file Presentation_1.pdf]

Supplementary material

Microbial hydrogen sinks in the sand-bentonite backfill material for the deep geological disposal of radioactive waste

1. Text

1. Experimental set-up

1.1.1. Reactor filling

All tubes were amended with fine mesh commercial organza fabric (Stoffe.de, fabfab GmbH, Schenefeld, Germany) to preclude the movement of sand or bentonite particles into the tubes. Pretests showed that this synthetic fiber, made out of 50% polyester and 50% polyamide was stable under the experimental conditions. The reactor was placed upside down for filling. Coarse sand was poured to reach 1.5 cm height, followed by 850 g of homogeneous sand-bentonite mix and another 1.5 cm of the coarse sand for the bottom layer. The bottom lid was then put into place and the reactor carefully inverted.

1.1.2. Abiotic control

As an abiotic control (referred to as NC), we used sterilized (γ-irradiated) Wyoming bentonite mixed with sterile quartz sand (autoclaved 80 minutes at 120°C). The sand bentonite was saturated with sterile artificial porewater (autoclaved 80 minutes), resembling the composition of Opalinus Clay porewater (Pearson et al., 2003). In contrast to the reactors, no flow-through system was adopted, but a volume of up to 5 mL of artificial porewater was added daily to compensate for evaporation losses within the dry atmosphere of the glovebox. After 30 days, the abiotic control was sampled for the same analyses as the other reactors. No genomic DNA (gDNA) was found nor could the 16S rRNA gene be amplified. Thus, NC could be used as a control for mineralogy study without the influence of microbial activity.

1.2. **Opalinus Clay formation borehole drilling**

A dedicated borehole was drilled in early May 2015 from the gallery at the Mont Terri Underground Rock Laboratory, with a 47° angle inclination into the ceiling of the gallery to a depth of 16.3 meters, perpendicularly to the bedding of the Opalinus Clay formation. Opalinus Clay mineralogy and porewater chemistry has been previously described (Pearson, 2002). The borehole is located at the main fault, at the transition from shaly to the sandy facies. To prevent contamination, all drill rods were cleaned with soap and water, rinsed with water and disinfected with 70% (v/v) ethanol that was allowed to evaporate prior to drill rod use. During handling, emphasis was put on keeping the rods as clean as possible, e.g., by usage of 70% (v/v) ethanol-treated nitrile gloves. After borehole drilling and packer insertion, the borehole and the sampling lines were flushed and pressurized with sterile-filtered argon gas to restore anaerobic conditions and minimize the disturbance to the indigenous microbial community developing in the space created.

2. Analytical methods

2.1. Opalinus Clay formation borehole microbial characterization

The microbial community of the porewater was investigated by DNA extraction and amplification of the V3-V4 region of the 16S rRNA gene. Triplicates borehole porewater samples (200 mL) were directly filtered using a sterile, DNA-free (autoclaved for 80 min), 0.2 μm polycarbonate filter membrane (Isopore Membrane hydrophilic polycarbonate, Merck

Millipore, Darmstadt, Germany) in a vacuum filter-tower setup (Nalgene® Nunc International, Rochester, New York, USA). The biomass retained on the filters were preserved in LifeGuard Soil Preservation solution (Qiagen NV, Venlo, The Netherlands), stored at -20°C until subjected to a modified phenol-chloroform-isoamyl alcohol DNA extraction protocol, based on (Bagnoud et al., 2016). Modifications applied were described by (Bell et al., 2018). Additionally, the polycarbonate filter remained in the test tubes until the addition of phenol. This treatment results in its solubilization, which potentially increases the DNA yield. The DNA cleanup procedure was performed with synthetic linearized polyacrylamide (GenElute™-LPA, MilliporeSigma, Merck KGaA, Darmstadt, Germany) rather than naturally sourced glycogen. Subsequent DNA analysis steps are similar to the reactor samples as described above. The borehole microbiome is described in **Table S2**.

2.2. qPCR calibration curve

qPCR was performed using a pGEM-T plasmid (Promega Corp. Madison, Wisconsin, U.S.A.) containing a E. coli 16S rRNA gene copy as a standard. The 6-point standard curve was obtained from 10^7 copies per µL, down to 100 copies per µL in ten-fold dilution steps. The amplification was carried out on a LightCycler® 96 Instrument (F. Hoffmann-La Roche AG, Basel, Switzerland) using LightCycler® 480Multiwell plates (96-well, white, F. Hoffmann-La Roche AG, Basel, Switzerland). For this system, the fluorescence threshold was determined to be at 0.404, which indicates the onset of an exponential increase of the fluorescence signal based on the amplification of a targeted DNA sequence. The amplification, barcoding and 16S rRNA library generation was carried out as suggested from Zymo Research Corp. PCR efficiencies were calculated using LinRegPCR (Ruijter et al., 2009), and the 16S rRNA gene copy number for the samples on each plate were calculated based on the standard curve method with the standard-values obtained from the same plate (Brankatschk et al., 2012).

2.3. Heatmaps

The heat maps were obtained with ampvis2, the number of reads is presented. ASVs are aggregated by genus, if the corresponding genus is not identified the lowest taxonomic rank is indicated. The 25 most abundant ASVs are shown and the 'remaining taxa' category contains taxa not in the top 25. Only genera with at least 0.1% relative abundance were considered. Because DNA sorbs to bentonite, equipment samples have greater number of reads than sand-bentonite samples. Thus, they were analyzed separately.

2.4. X-ray fluorescence analysis

The laser gun was equipped with a Rh micro-focus X-ray tube at 45 kV acceleration and 1 mA current and an Apollo XRF-ML50 Silicon Drift Detector. A 25 µm titanium foil filter was applied to reduce the background signal in the energy range of the sulfur K-edge and the spectra were recorded for 10 seconds at each spot. The spot size was 30 µm. Overview maps had a scanning step size of 600 µm.

3. Sulfate in the sand-bentonite matrix

Reactors contain 170 g of bentonite, with a water content of 8%. The molar quantity of sulfate that can leach resulting from gypsum dissolution was calculated to be:

$$n_{sulfate} = \frac{m_{bentonite}}{1 + w} * x_{gypsum} + \frac{1}{M_{sulfate}} = 8.5\ mmol$$

$With$:

$m_{bentonite}$: $the\ mass\ of\ bentonite\ in\ one\ reactor$
w: $the\ water\ content\ of\ the\ initial\ bentonite$
x_{gypsum}: $the\ mass\ percent\ of\ gypsum\ in\ bentonite\ MX80$
$M_{sulfate}$: $the\ molar\ mass\ of\ sulfate$

4. Volume of backfill required

4.1. Hydrogen-oxidation rate per volume of backfill

$$n_{H_2,backfill}[\frac{\mathrm{mol}}{\mathrm{day.\,m_{backfill}^3}}] = n_{\mathrm{H_2},water}[\frac{\mathrm{mol}}{\mathrm{day.\,m_{water}^3}}] * \frac{V_{water}}{V_{backfill}}$$

$With$:
$n_{H_{2_{water}}}$: $the\ hydrogen - oxidation\ rate\ per\ volume\ of\ water$
V_{water}: $the\ volume\ of\ water\ in\ the\ porous\ medium\ (i.e.the\ volume\ of\ voids\ in\ case\ of\ saturated\ m$
$V_{backfill}$: $the\ volume\ of\ backfill$

Here we consider: $V_{water} = 343\ cm^3\ and\ V_{backfill} = 733\ cm^3$. These values were estimated from the mass of sand/bentonite (850 g) and the volume of coarse sand (200 cm^3), using dry densities from (Manca, 2015), and considering saturated material.

4.2. Volume of backfill required

$$V_{backfill} = \frac{H_{2,total}}{H_{2,volumetric\ backfill\ capacity}} = \frac{H_{2,total}}{n_{H_2,backfill} * 1{,}000{,}000\ years}$$

Here we consider

(a) a confinement duration of 1,000,000 years (Allan Hedin, 1997),
(b) a total production of H_2 during repository lifetime of 25 Mm^3 (900 x 10^6 mol) (Diomidis et al., 2016; O.X. Leupin, 2016).

2. Figures

2.1. Experimental set-up

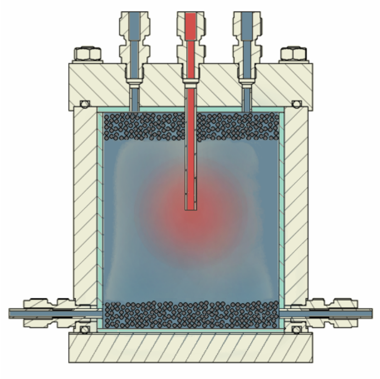

Figure S1 - A stainless steel reactor (beige), equipped with an inner Plexiglas cylinder (turquoise) filled with an 80/20% (w/w dry mass) mixture of sand-bentonite. Additionally, layers of coarse sand were included in the top and bottom part of the reactor. The sand and the sand-bentonite mixture are saturated (blue) with Opalinus Clay porewater from the borehole through two water inlets at the bottom. Daily pulses of hydrogen gas were applied to the middle (red) via a titanium tube in the center of the sand-bentonite core. Water outlets on the top enabled the porewater to follow a controlled pressure gradient. The dark blue area indicates the expected preferential water flow path along the contact surfaces of the Plexiglas cylinder.

2.2. Sand-bentonite color after incubation

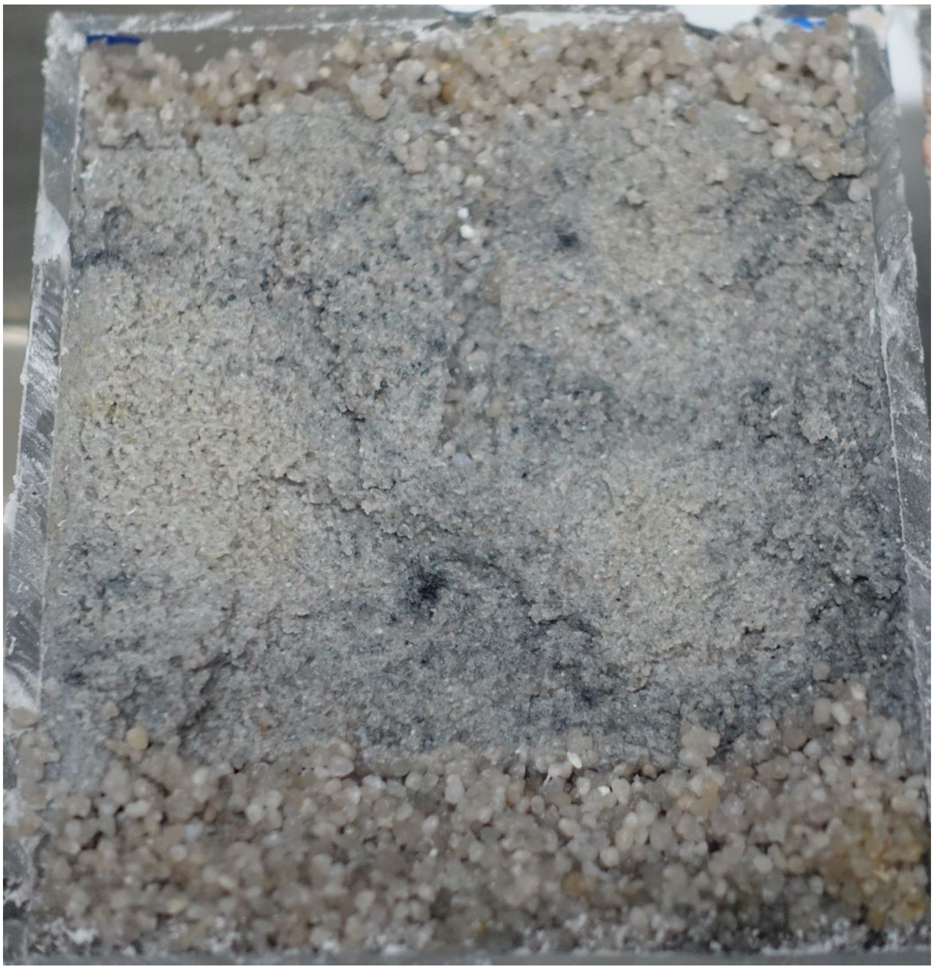

Figure S2 - Reactor interior in anxoic condtitions: sand-bentonite after incubation (reactor 3) but prior to sampling for DNA. The bentonite appears darker at the bottom and closer to the Plexiglas cylinder. Two black spots can be observed at the lower middle and the upper middle-right part.

2.3. Flow rate and sulfate concentration

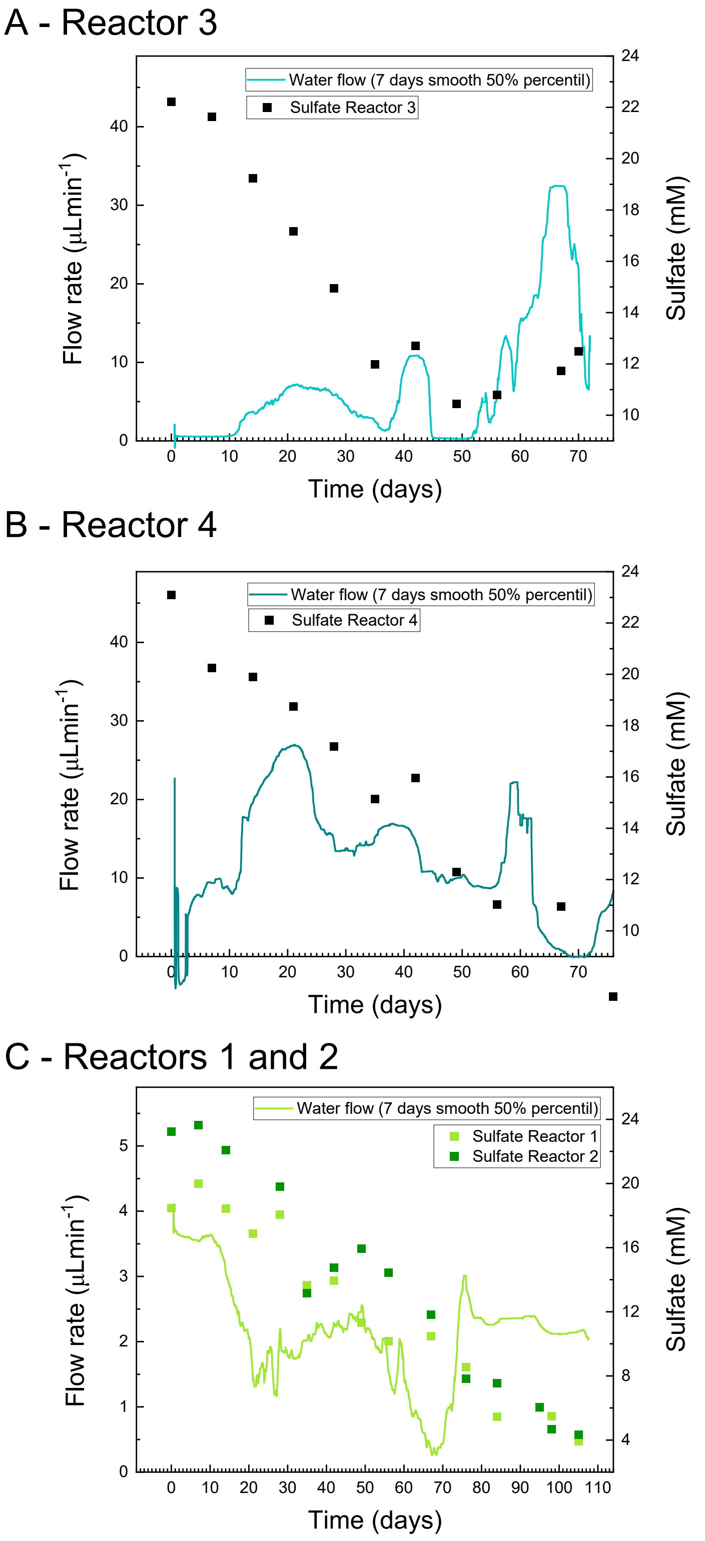

Figure S3 – Time evolution of flow rate (full line) and sulfate concentration (points) of porewater entering the reactors. Flow rate is presented as a rolling average over 7 days, using a cutoff at the 50% percentile. This is done because samples for sulfate concentration were taken every 7 days. (**A**) Reactor 3, (**B**) Reactor 4, (**C**) Reactor 1 and 2. The scale on the y-axis is 10-fold lower for (**C**).

2.4. Methane detection

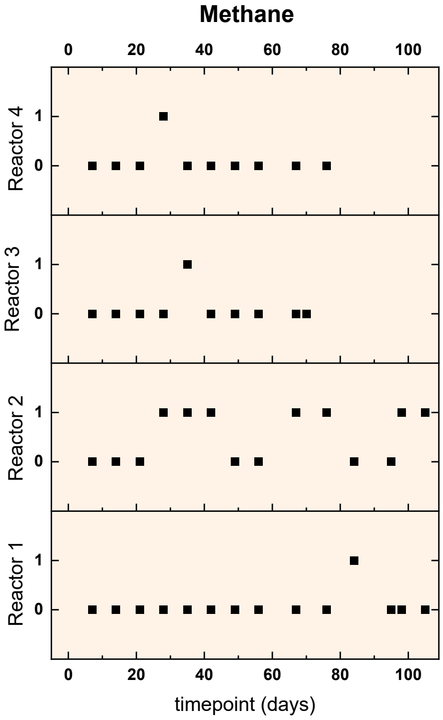

Figure S4 – Detection of methane in the gas samples of the four reactors. 1 indicates that methane was detected in the sample, and 0 that it was not.

2.5. X-ray fluorescence ratios

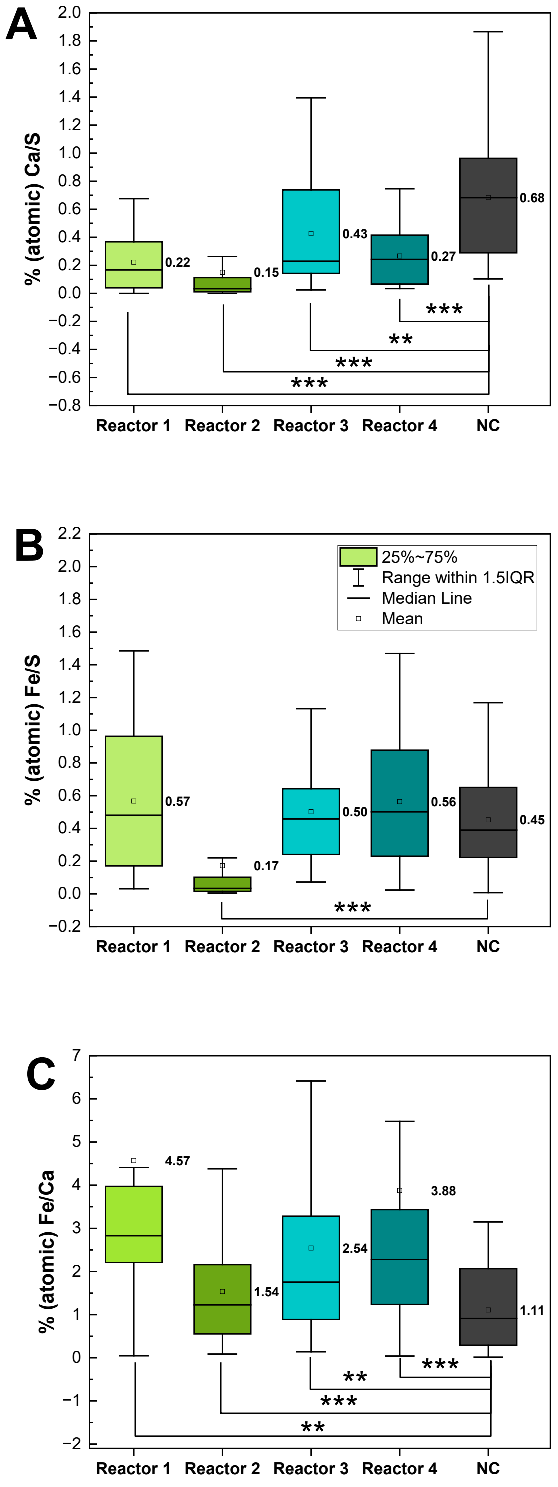

Figure S5 – Results of elemental counting and mapping using XRF, at accumulated sulfur spots (> 10% w): (**A**) ratio of atomic percent of calcium to sulfur; (**B**) ratio of atomic percent of iron to sulfur; (**C**) ratio of atomic percent of iron to calcium. Boxplot were built using Origin Pro 2022b, NC refers to the abiotic control. Only points with sulfur atomic ratio above 10% were considered. T-tests were achieved (Excel Analysis ToolPak, previously testing equality of variance with F-test) to assess statistical significance of the minerals shifts between biotic reaction and abiotic control (*: < 0.05, **: < 0.01, ***; < 0.001, **Table S12**).

2.6. XANES maps

Small samples of the sand-bentonite matrix from reactors 2 and 3 were analyzed using bright, coherent synchrotron X-ray radiation at SSRL. MicroXRF images of iron and sulfur were produced to identify spots with iron sulfide or sulfur accumulation. Iron speciation analysis was performed on each pixel of the maps and on several spots outside the mapped area.

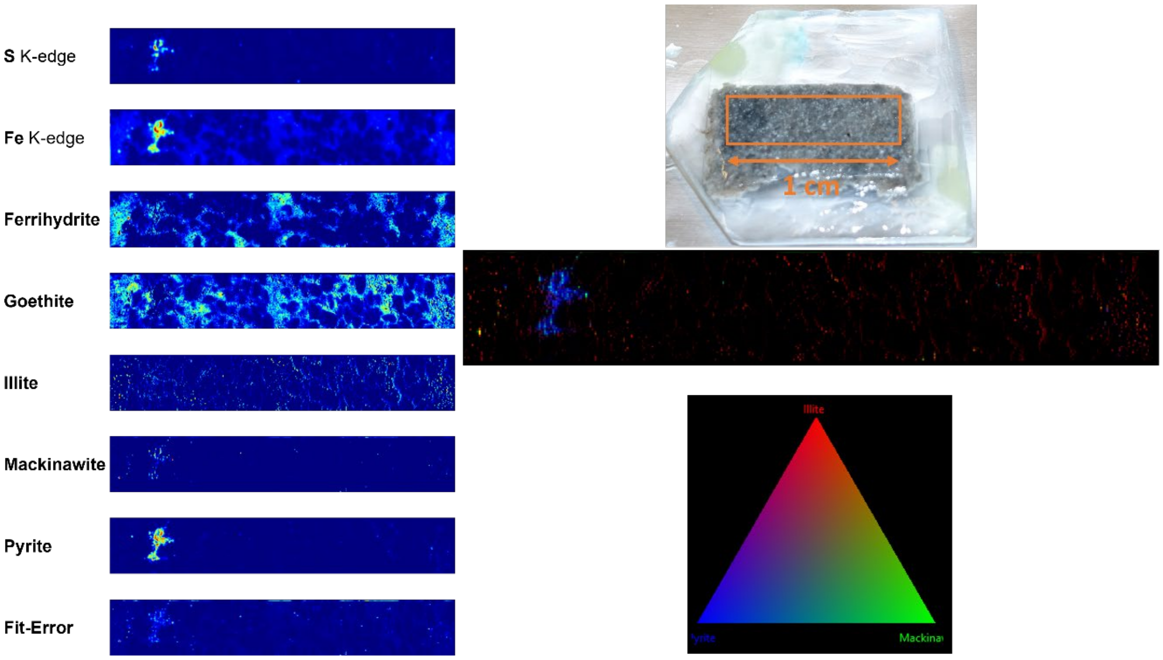

Figure S6 – Maps of iron and sulfur, and fits, obtained from the orange area in reactor 2, a spot of pyrite is observed, with a size of approximatively 1mm^2

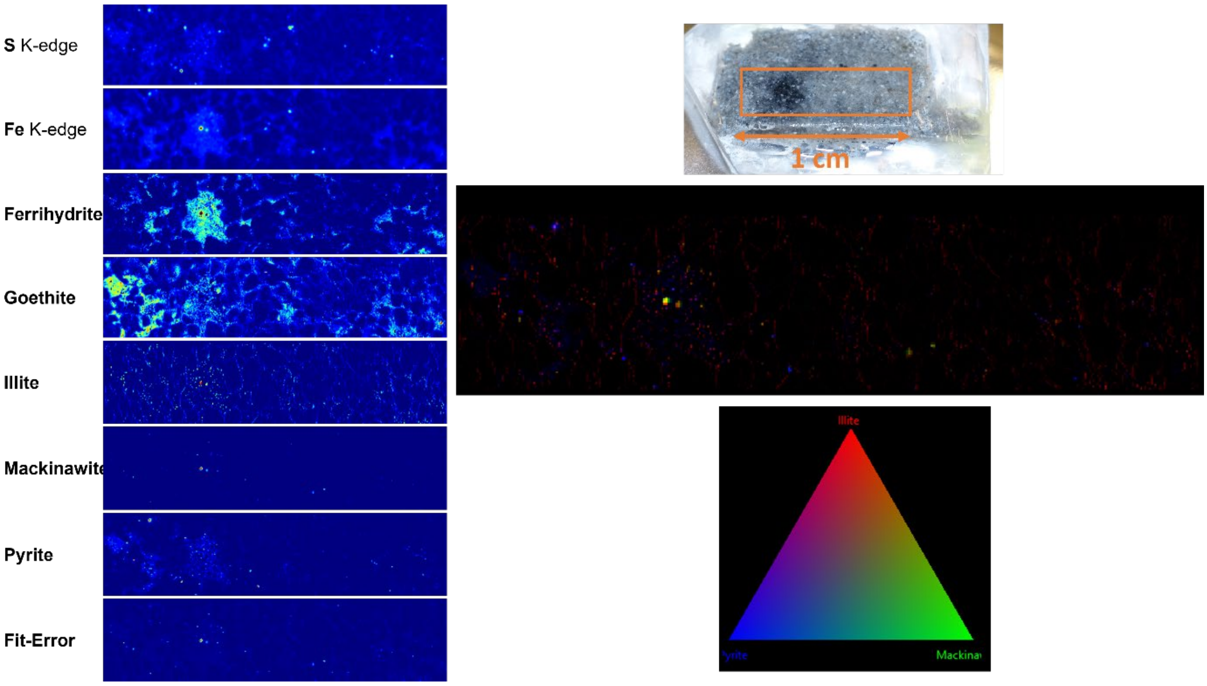

Figure S7 – Maps of iron and sulfur, and fits, obtained from the orange area in reactor 3, a spot of ferrihydrite is observed, with a size of approximatively 3mm^2

2.7. Results of biomass quantification using qPCR

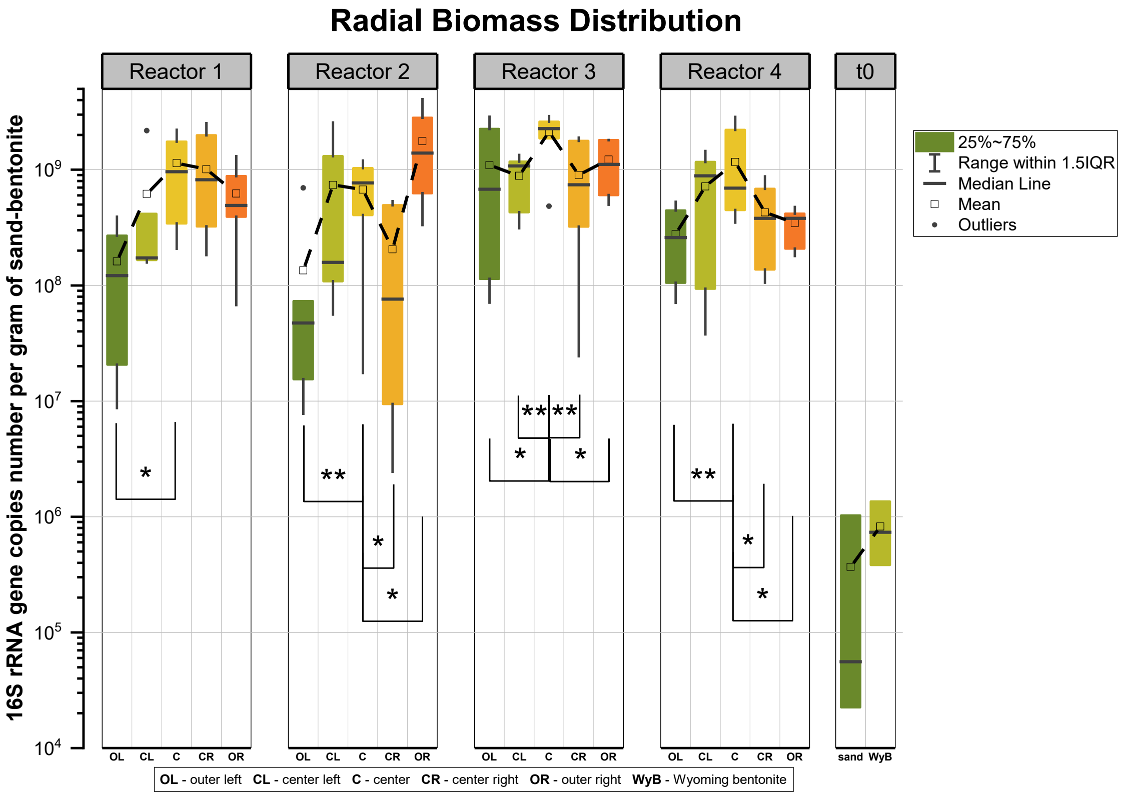

Figure S8 – Radial biomass distribution, based on 16S rRNA gene copies number per gram of sand-bentonite. On the left, results for initial time point (t0) for dry sand and Wyoming bentonite are presented. T-tests were achieved (Excel Analysis ToolPak, previously testing equality of variance with F-test) to assess statistical significance of the patterns in biomass distribution within each reactor (*: < 0.05, **: < 0.01, ***; < 0.001 **Table S11**).

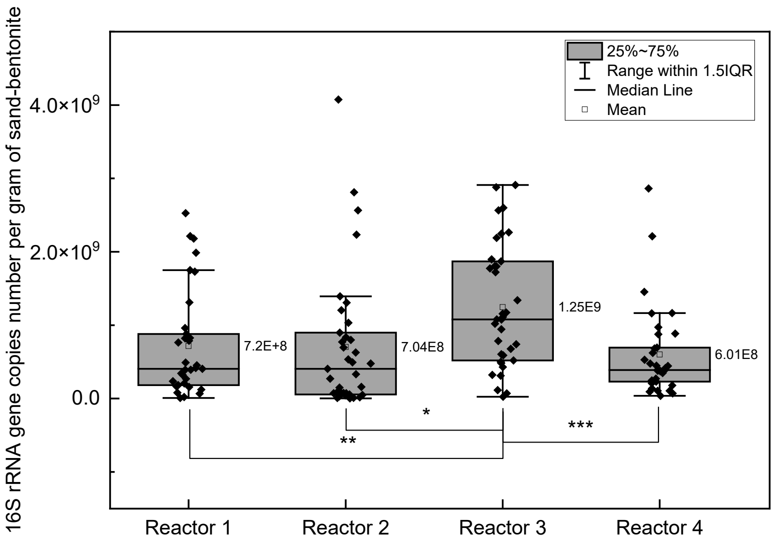

Figure S9 – 16S rRNA gene copies number per gram of sand-bentonite for all 4 reactors. Boxplots were built with Origin2022. T-tests were achieved (Excel Analysis ToolPak, previously testing equality of variance with F-test) to assess the statistical significance of differences in average biomass concentration across all 4 reactors (*: < 0.05, **: < 0.01, ***; < 0.001 **Table S10**).

2.8. Principal component analysis

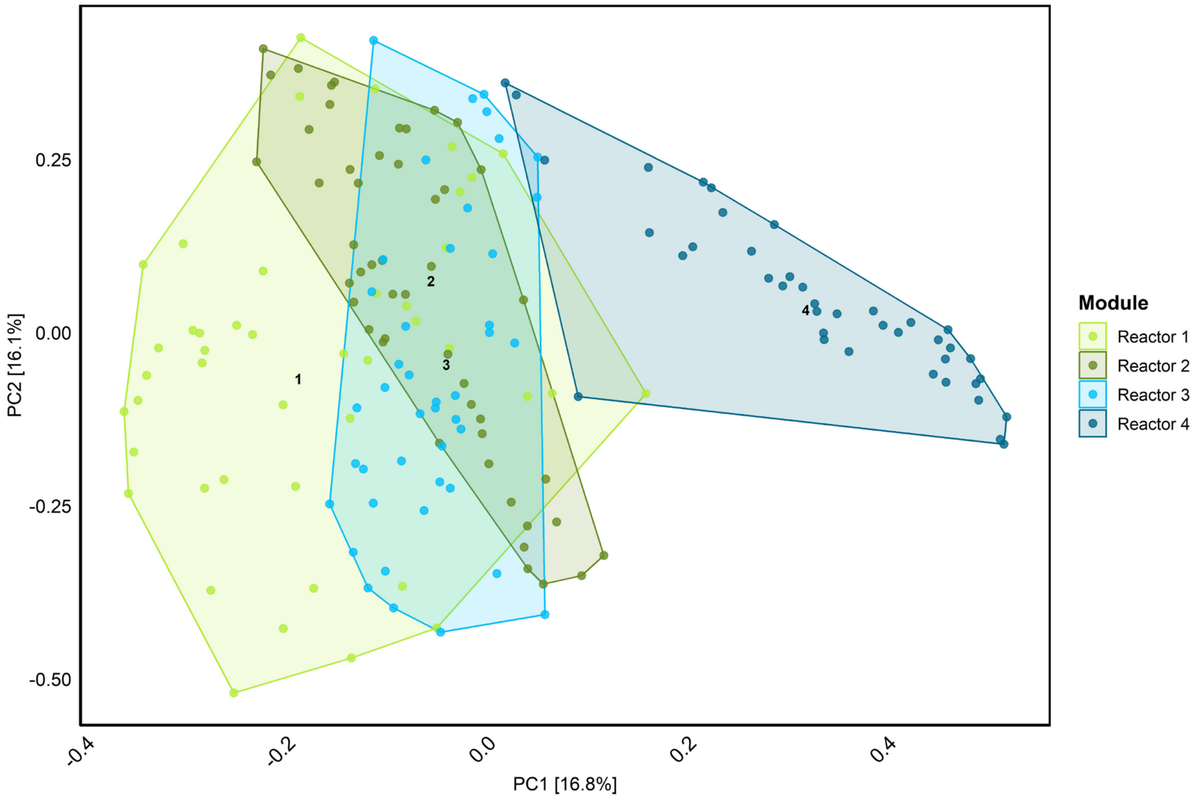

Figure S10 – Principal Component Analysis of the 16S rRNA sequences after aggregation by taxonomic affiliation (using Hellinger transformation). Each point represents a sample. The difference with **Figure 3** is that the equipment samples are included for each reactor, which results in overlapping of the clouds.

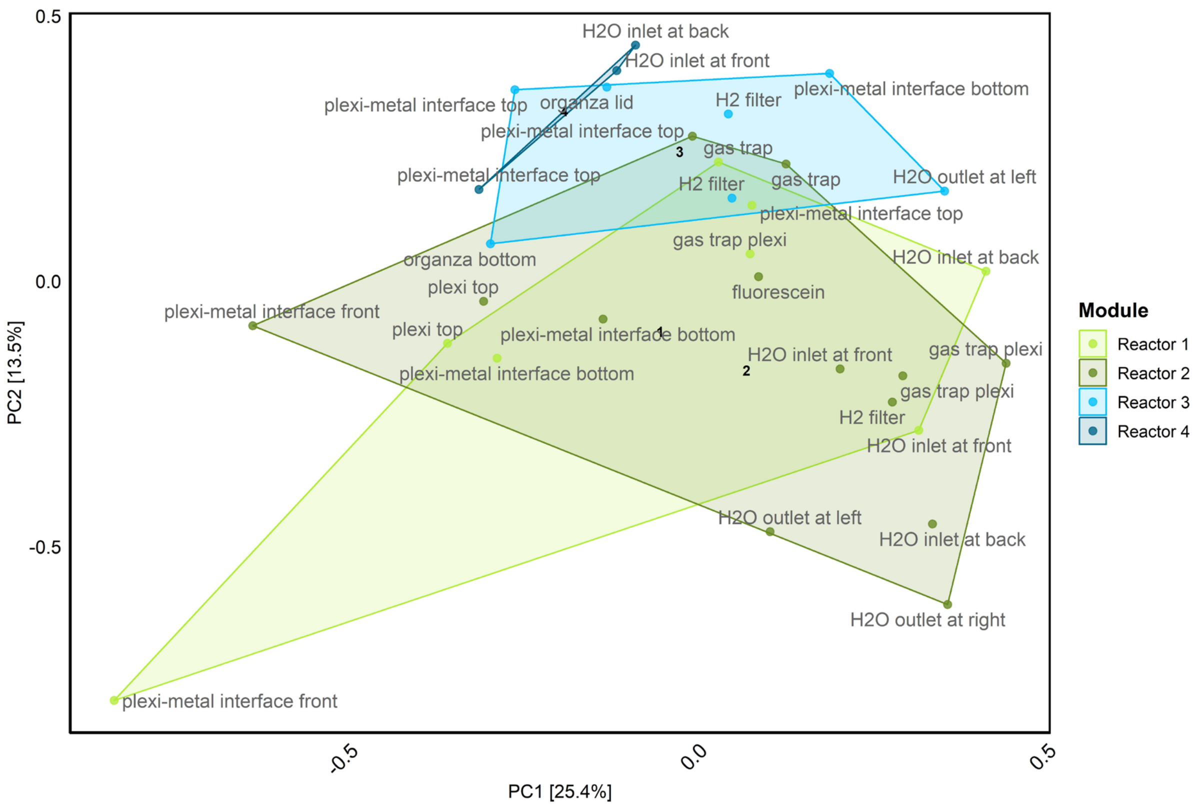

Figure S11 – Principal Component Analysis of the 16S rRNA sequences of equipment samples after aggregation by taxonomic affiliation (using Hellinger transformation). Each point represents a sample.

3. Tables

Table S1 – Wyoming bentonite MX-80 mineralogical composition, based on (Karnland et al., 2006)

**Mineral phase**	**Weight [%]**
Montmorillonite	83.50
Illite	0.70
Anatase	0.20
Calcite	0.20
Cristobalite	0.40
Goethite	0.20
Gypsum	0.90
Hematite	0.10
Lepidocrocite	0.70
Magnetite	0.10
Microcline	0.80
Muscovite	2.80
Orthoclase	0.70
Plagioclase	2.90
Pyrite	0.60
Quartz	2.80
Tridymite	1.90

Table S2 - Heat map of ASV relative abundance obtained from 16S rRNA gene sequencing from filtered borehole porewater microorganisms within the borehole BMA-A1 that served as natural water source for the experiment. Five time points were taken between March 2017 and September 2019.

	2017-03		2017-05	2017-10	2018-04		2019-09	
Proteobacteria; Pseudomonas	42.4	48.8	65.4	30.7	16.8	24.9	48.8	47.7
Firmicutes; f__Paenibacillaceae_1_ASV21	0.5	0.8	2.9	43.7	40	36.8	13.9	12.7
Firmicutes; Desulfosporosinus	2.2	4.2	1	7.3	17	13.5	1.4	1.5
Firmicutes; o__Clostridiales_ASV28	8	7.7	6.8	1.7	3.5	3.5	1.6	2
Firmicutes; o__Clostridiales_ASV42	3.7	4.8	3.4	8.4	5.5	2.7	1.8	1.6
Firmicutes; f__Peptococcaceae_2_ASV10	4.8	5.7	4.9	1.9	1.6	2.5	4.3	4
Proteobacteria; Limnobacter	8.9	6.7	3	1.1	0.7	1	2.2	2.1
Firmicutes; Gracilibacter	3.1	4.3	3.1	2.3	0.7	1.7	1.6	1.6
Firmicutes; p__Firmicutes_ASV19	0	0	0	0	0	0.5	6.5	7.4
Proteobacteria; Phenylobacterium	0	0.1	0	0	4.5	4.3	1.2	2.3
Firmicutes; f__Peptococcaceae_1_ASV57	1.4	1.6	1.5	0.4	2.2	1.5	1.3	1.6
Firmicutes; f__Ruminococcaceae_ASV103	6.8	4	0.3	0	0	0	0	0
Firmicutes; f__Peptococcaceae_2_ASV43	0.2	0.5	0.3	0.2	1.7	0.7	1.8	1.7
Proteobacteria; f__Oxalobacteraceae_ASV35	0.3	0.5	0.1	0.1	0.3	0.5	2.4	2.1
Firmicutes; Desulfocucumis	0	0	0.8	0.7	0.7	0.5	1.7	1.5
Firmicutes; f__Ruminococcaceae_ASV240	0	0.1	0.3	0.1	0	0	2.6	2.6
Proteobacteria; Brevundimonas	1.8	1.2	1.4	0	0	0.2	0.3	0.5
Planctomycetes; f__Isosphaeraceae_ASV283	1.6	1.9	0.1	0	0	0.5	0.2	0.1
k__Bacteria_ASV132; k__Bacteria_ASV132	0	0.1	0.1	0.1	0.8	0.5	1.1	1.1
Firmicutes; c__Clostridia_ASV11	0.2	0.5	0.4	0.2	0.4	0.2	0.8	0.9
Proteobacteria; Caulobacter	2.4	0.6	0.1	0	0	0	0	0
Firmicutes; Acetivibrio	1.8	0.5	0.2	0.1	0	0	0.1	0.1
Firmicutes; f__Ruminococcaceae_ASV425	0.1	0	0	0	0	0	1.2	1.3
Firmicutes; o__Clostridiales_ASV66	0	0.9	0.3	0.2	0	0.4	0.3	0.4
Proteobacteria; f__Xanthobacteraceae_ASV421	0.6	0.6	0.1	0	0.5	0.2	0	0
Remaining taxa (117)	9.1	3.7	3.5	0.8	3.2	3.3	2.9	3.3

% Read Abundance
60
40
20

Table S3 – Opalinus Clay porewater composition from March 2017 to September 2019

	15.03.201	15.05.201	31.10.20217	11.04.2021	24.09.2019	mean	SD
Eh vs. SHE	-267.9	-261.6	-316.6	-366.5	-332.5	-309.0	39.6
pH	6.4	7.3	8.0	7.1	7.2	7.2	0.5
DOC	6.80	9.71	7.01	4.76	19.44	9.54	5.19
DIC	63.95	112.85	58.87	54.60	47.35	67.52	23.31
Na^{+} [mM]	198.400	198.567	152.754	187.955	217.696	191.07	21.432
NH_4^{+} [mM]	1.619	5.866	-	2.262	0.552	2.575	2.061
K^{+} [mM]	1.835	1.298	1.642	1.572	1.440	1.557	0.182
Mg_2^{+} [mM]	13.989	13.937	13.875	13.788	15.063	14.130	0.471
Ca_2^{+} [mM]	12.357	12.391	12.575	12.469	11.990	12.356	0.198
F^{-} [mM]	-	-	-	-	-	-	-
Cl^{-} [mM]	242.891	245.678	241.992	231.202	245.316	241.41	5.295
NO_2^{-} [mM]	-	-	-	0.304	-	0.304	0.121
SO_4^{2-} [mM]	15.659	15.226	14.897	14.682	14.042	14.901	0.541
NO_3^{2-}	-	0.559	-	1.560	-	1.060	0.608
PO_4^{2-} [	302	-	3	-	-	153	121
Al [µM]	-	-	-	-	-	-	-
Co [µM]	-	-	-	-	-	-	-
Cr [µM]	-	-	-	-	-	-	-
Cu [µM]	-	-	-	-	-	-	-
Fe [µM]	-	7	-	-	29	18	
Mn [µM]	-	2	-	-	-	2	
Ni [µM]	-	-	-	-	2	2	
Si [µM]	-	63	101	109	-	91	47
Sr [µM]	-	186	395	382	192	289	146
Zn [µM]	-	-	-	-	12	12	5

Table S4 – Detail of the sulfate rate linear regression for each reactor (using Origin Pro 2022b)

	Intercept	Intercept	Slope	Slope	Statistics
	**Value**	**Standard Error**	**Value**	**Standard Error**	**Adjusted R-square**
**Reactor 1**	20.07	0.55	-0.15	0.009	0.955
**Reactor 2**	23.88	0.78	-0.192	0.012	0.949
**Reactor 3**	20.80	1.16	-0.163	0.028	0.771
**Reactor 4**	22.50	0.49	-0.191	0.011	0.966

Table S5 – Statistical analysis of the XRF atomic counting on one half of the reactors (spot size of 30 µm, step size of 600 µm) compared to no-H_2 control. Only points with sulfur atomic percent above 10% were considered, and the corresponding number (n) is indicated for each sample.

	**Reactor 1 (n = 23)**		**Reactor 2 (n = 225)**		**Reactor 3 (n = 40)**		**Reactor 4 (n = 18)**	
	F-test	t-test	F-test	t-test	F-test	t-test	F-test	t-test
**%atomic Calcium:Sulfur**								
**NC (n = 34)**	0.0001	4e-7	0.0026	6e-9	0.3850	0.0039	0.0034	9e-6
**%atomic Iron:Sulfur**								
**NC (n = 34)**			0.2773	2e-6				
**%atomic Iron:Calcium**								
**NC (n = 34)**	4e-18	0.0092	0.0166	0.0138	2e-7	0.0008	10e-15	0.0169

Table S6 - Results of phase identification using XANES, and LCF with Athena, sulfur speciation. Phases with non-null weight are indicated. R-factor and reduced χ^2 are statistical indicators of the goodness of the fit.

Sample		Gypsum		Mackinawite/Pyrrhotite		Pyrite		r-factor	Reduced χ^2
Reactor 4	1	-	-	**0.2656**	0.0153	**0.7028**	0.0163	0.0105	0.0023
	2	0.0138	0.0323	**0.2528**	0.0231	**0.6559**	0.0221	0.0213	0.0049
		-	-	0.1647	0.027	**0.7795**	0.0288	0.0322	0.0081
	3	-	-	**0.2813**	0.0144	**0.689**	0.0153	0.0098	0.0021
	4	-	-	**0.2415**	0.0193	**0.7198**	0.0193	0.0144	0.0033
Reactor 3	1	**0.4799**	0.1007	**0.2725**	0.0712	0.0053	0.0704	0.1515	0.0713
		0.0748	0.0262	**0.2797**	0.0187	**0.5819**	0.0187	0.0148	0.0033
	2	-	-	**0.2888**	0.017	**0.6761**	0.018	0.0127	0.0029
	3	-	-	**0.2284**	0.0134	**0.7447**	0.0134	0.0077	0.0016
Reactor 1	1	-	-	**0.2171**	0.0231	**0.6726**	0.0872	0.0242	0.0058
		**0.7831**	0	0.1355	0	0.0814	0	0.198	0.1937
	2	-	-	**0.2574**	0.0161	**0.6395**	0.087	0.0132	0.003
	3	-	-	**0.2977**	0.0144	**0.6016**	0.0863	0.0101	0.0021
Reactor 2	1	-	-	**0.2703**	0.0363	**0.6617**	0.0317	0.0431	0.0097
	2	-	-	0.1501	0.1378	**0.5751**	0.1371	0.5507	0.3186
	3	0.0678	0.043	**0.8462**	0.043	-	-	0.2568	0.0648
	4	-	-	0.103	0.0318	**0.8333**	0.0318	0.0384	0.01
NC	1	**1**	0	-	-	-	-	0.2969	0.3441
	2	**0.4809**	0.0383	0.1514	0.071	0.1491	0.1093	0.165	0.0673
	3	**0.8696**	0	0.1304	0	-	-	0.0881	0.057

Table S7 - Results of phase identification using XANES, and LCF with Athena, iron speciation. Phases with non-null weight are indicated. R-factor and reduced χ^2 are statistical indicators of the goodness of the fit.

Sample		Fe(III) bentonite		Ferrihydrite		Illite		Pyrite		Mackinawite		Goethite		r-factor	reduced χ^2
Reactor 2	**2**	**0.887**	0.024	0.064	0.025	-	-	-	-	-	-	-	-	0.0039	0.0011
	**3**	**1.000**	0.000	-	-	-	-	-	-	-	-	-	-	0.0040	0.0011
	**6**	0.162	0.013	0.082	0.023	-	-	**0.371**	0.013	**0.320**	0.017	-	-	0.0060	0.0010
	**4**	**0.611**	0.032	0.021	0.018	**0.296**	0.021	-	-	-	-	-	-	0.0015	0.0004
	**7**	**0.801**	0.036	-	-	0.125	0.038	-	-	-	-	-	-	0.0064	0.0023
	**8**	**0.919**	0.000	0.040	0.000	0.041	0.000	-	-	-	-	-	-	0.0084	0.0028
Reactor 4	**9**	0.173	0.011	-	-	0.075	0.010	**0.404**	0.012	**0.302**	0.012	-	-	0.0004	0.0001
	**10**	**0.300**	0.021	0.092	0.012	**0.560**	0.015	-	-	-	-	-	-	0.0007	0.0002
	**11**	0.071	0.031	**0.558**	0.055	-	-	0.056	0.032	0.155	0.041	-	-	0.0034	0.0010
	**12**	0.064	0.000	**0.789**	0.000	-	-	-	-	0.147	0.000	-	-	0.0516	0.0196
	**13**	**0.592**	0.052	**0.302**	0.054	-	-	-	-	-	-	-	-	0.0228	0.0056
	**14**	-	-	-	-	-	-	**0.395**	0.019	**0.568**	0.019	-	-	0.0016	0.0003
	**15**	-	-	**0.446**	0.030	-	-	-	-	0.069	0.021	**0.416**	0.017	0.0043	0.0009
	**16**	**0.207**	0.056	**0.679**	0.058	-	-	-	-	-	-	-	-	0.0261	0.0064
	**17**	**0.920**	0.000	0.080	0.000	-	-	-	-	-	-	-	-	0.0090	0.0029
	**18**	**0.378**	0.027	0.025	0.015	**0.539**	0.018	-	-	-	-	-	-	0.0010	0.0003
Reactor 3	**19**	**0.611**	0.038	**0.311**	0.040	-	-	-	-	-	-	-	-	0.0118	0.0043
	**20**	0.120	0.000	**0.747**	0.000	-	-	-	-	0.133	0.000	-	-	0.0532	0.0235
	**21**	0.114	0.024	0.158	0.042	-	-	**0.320**	0.024	**0.287**	0.031	-	-	0.0022	0.0004
	**25**	**0.828**	0.000	0.159	0.000	0.013	0.000	-	-	-	-	-	-	0.0138	0.0057

Table S8 – Average relative abundance of top ASVs in the 35 sand-bentonite samples in reactors 1 and 2. Relative abundance was calculated for each ASV from the average number of reads in the reactors from which the number of reads of the ASV in the extraction controls was subtracted. Sulfate reducing bacteria are highlighted in blue and methanogens in green.

ASV	Relative abundance (%)	ASV	Relative abundance (%)
**Reactor 1**		**Reactor 2**	
Proteobacteria; Pseudomonas	16.6	Bacteroidetes; Sunxiuqinia	19.1
Proteobacteria; c__Deltaproteobacteria_ASV6	13.7	Proteobacteria; Pseudomonas	17.6
Proteobacteria; f__Desulfuromonadaceae_ASV7	11.7	Proteobacteria; c__Deltaproteobacteria_ASV13	13.5
Euryarchaeota; Methanosarcina	7.1	Proteobacteria; f__Desulfuromonadaceae_ASV7	12.3
Bacteroidetes; Sunxiuqinia	6.4	k__Bacteria_ASV16; k__Bacteria_ASV16	8.7
Proteobacteria; Desulfocurvibacter	5.5	Proteobacteria; Desulfocurvibacter	7.3
Firmicutes; Symbiobacterium	5.3	Euryarchaeota; Methanosarcina	5.2
Firmicutes; f__Peptococcaceae_2_ASV10	4.9	Firmicutes; Symbiobacterium	4.2
Proteobacteria; f__Desulfobacteraceae_ASV3	4.8	Firmicutes; Desulfotomaculum	3.4
Proteobacteria; f__Desulfuromonadaceae_ASV33	4.6	Chloroflexi; f__Anaerolineaceae_ASV24	2.4
Firmicutes; Gracilibacter	3.0	Firmicutes; p__Firmicutes_ASV19	2.2
Firmicutes; c__Clostridia_ASV11	2.1	Proteobacteria; f__Desulfobacteraceae_ASV3	1.9
Proteobacteria; f__Desulfuromonadaceae_ASV14	0.9	Firmicutes; Gracilibacter	1.4
Firmicutes; Desulfallas	0.6	Firmicutes; Desulfallas	0.8
Proteobacteria; Pelomonas	0.0	Proteobacteria; Pelomonas	0.0
Remaining taxa (2508)	12.9	Remaining taxa (677)	0.0
**Sulfate reducing bacteria**	**46.9**	**Sulfate reducing bacteria**	**38.7**

Table S9 – Average relative abundance of top ASVs in the 35 sand-bentonite samples in reactors 3 and 4. Relative abundance was calculated for each ASV from the average number of reads in the reactors from which the number of reads of the ASV in the extraction controls was subtracted. Sulfate-reducing bacteria are highlighted in blue and methanogens in green.

ASV	Relative abundance (%)	ASV	Relative abundance (%)
**Reactor 3**		**Reactor 4**	
Proteobacteria; f__Desulfobacteraceae_ASV3	16.5	Bacteroidetes; Sunxiuqinia	22.4
Bacteroidetes; Sunxiuqinia	16.3	Proteobacteria; c__Deltaproteobacteria_ASV15	21.4
Proteobacteria; c__Deltaproteobacteria_ASV6	11.9	Proteobacteria; Pseudomonas	11.6
Proteobacteria; Pseudomonas	10.3	Firmicutes; p__Firmicutes_ASV22	8.1
Proteobacteria; f__Desulfuromonadaceae_ASV7	9.1	k__Bacteria_ASV29; k__Bacteria_ASV29	7.9
Firmicutes; c__Clostridia_ASV17	8.4	Firmicutes; f__Peptococcaceae_2_ASV10	7.2
Firmicutes; Desulfotomaculum	7.5	Proteobacteria; Desulfocurvibacter	5.5
Firmicutes; c__Clostridia_ASV11	7.2	Proteobacteria; f__Desulfobacteraceae_ASV3	4.0
Firmicutes; Symbiobacterium	4.1	Firmicutes; Symbiobacterium	3.7
Proteobacteria; Desulfocurvibacter	3.8	Firmicutes; c__Clostridia_ASV17	3.2
Firmicutes; f__Peptococcaceae_2_ASV10	3.1	Firmicutes; c__Clostridia_ASV11	2.8
Firmicutes; Gracilibacter	1.1	Firmicutes; Desulfallas	1.9
Euryarchaeota; Methanosarcina	0.6	Firmicutes; Gracilibacter	0.3
Firmicutes; Desulfallas	0.1	Proteobacteria; Pelomonas	0.0
Proteobacteria; Pelomonas	0.0	Euryarchaeota; Methanosarcina	0.0
Remaining taxa (546)	0.0	Remaining taxa (581)	0.0
**Sulfate reducing bacteria**	**50.2**	**Sulfate reducing bacteria**	**42.2**

Table S10 - Heat map of ASVs relative abundances obtained from 16S rRNA gene sequencing from the four reactors. Equipment samples were not included, the kitome contribution was not removed.

	1	2	3	4
Pseudomonas; ASV1	11.1	15	10.5	13.8
Desulfocurvibacter; ASV2	8.4	10.4	9.1	9.1
Sunxiuqinia; ASV5	3.3	10.5	6.1	11.3
f__Desulfobacteraceae_ASV3; ASV3	5.4	3.9	11.6	7.9
c__Deltaproteobacteria_ASV6; ASV6	13.3	0	14.1	0
Methanosarcina; ASV4	9.4	8.9	3	1
Symbiobacterium; ASV8	2	3.8	6.9	8.5
f__Desulfuromonadaceae_ASV7; ASV7	6.4	8.3	5.1	0
f__Peptococcaceae_2_ASV10; ASV10	2.9	1	2.5	4.5
c__Clostridia_ASV11; ASV11	1.9	1.3	4	3
c__Deltaproteobacteria_ASV15; ASV15	0.1	0	0	9.8
Gracilibacter; ASV9	2.5	2.2	2.2	1.7
c__Deltaproteobacteria_ASV13; ASV13	0.4	7	0	0
c__Clostridia_ASV17; ASV17	1.2	0.7	2.9	1.9
f__Desulfuromonadaceae_ASV14; ASV14	4.7	0.3	0	0.9
k__Bacteria_ASV16; ASV16	0.7	4.5	0.8	0
p__Firmicutes_ASV22; ASV22	0	0	1.2	3.5
Desulfotomaculum; ASV23	0.4	1.1	1.8	0
k__Bacteria_ASV29; ASV29	0	0	0	3.2
Gracilibacter; ASV18	0.9	0.8	0.9	0.7
p__Firmicutes_ASV19; ASV19	0.6	1.6	0.5	0.2
Symbiobacterium; ASV27	2	0.5	0	0
Symbiobacterium; ASV26	2	0.5	0	0
Symbiobacterium; ASV37	0.2	0.4	0.8	0.9
Gracilibacter; ASV25	0.7	0.6	0.5	0.4
Remaining taxa (3831)	19.2	16.7	15.5	17.8

% Read Abundance: 15, 10, 5

Table S11 – Statistical analysis of the biomass (i.e., number of 16S rRNA gene copies per gram of sand-bentonite) in reactor 1, 2, and 4 compared to reactor 3 (Excel Analysis Toolpak). n = 35 samples were taken per reactor.

	**Reactor 1**		**Reactor 2**		**Reactor 4**	
	F-test	t-test	F-test	t-test	F-test	t-test
**Reactor 3**	0.1976	0.0036	0.2220	0.0218	0.0389	0.0003

Table S12 – Heat map of ASVs relative abundances obtained from 16S rRNA gene sequencing from the equipment (swabs etc.) taken on each reactor (1, 2, 3, 4), and from the controls (Kitome: DNA extraction kit, NTC: no template control well, Zymo Community: positive control well.

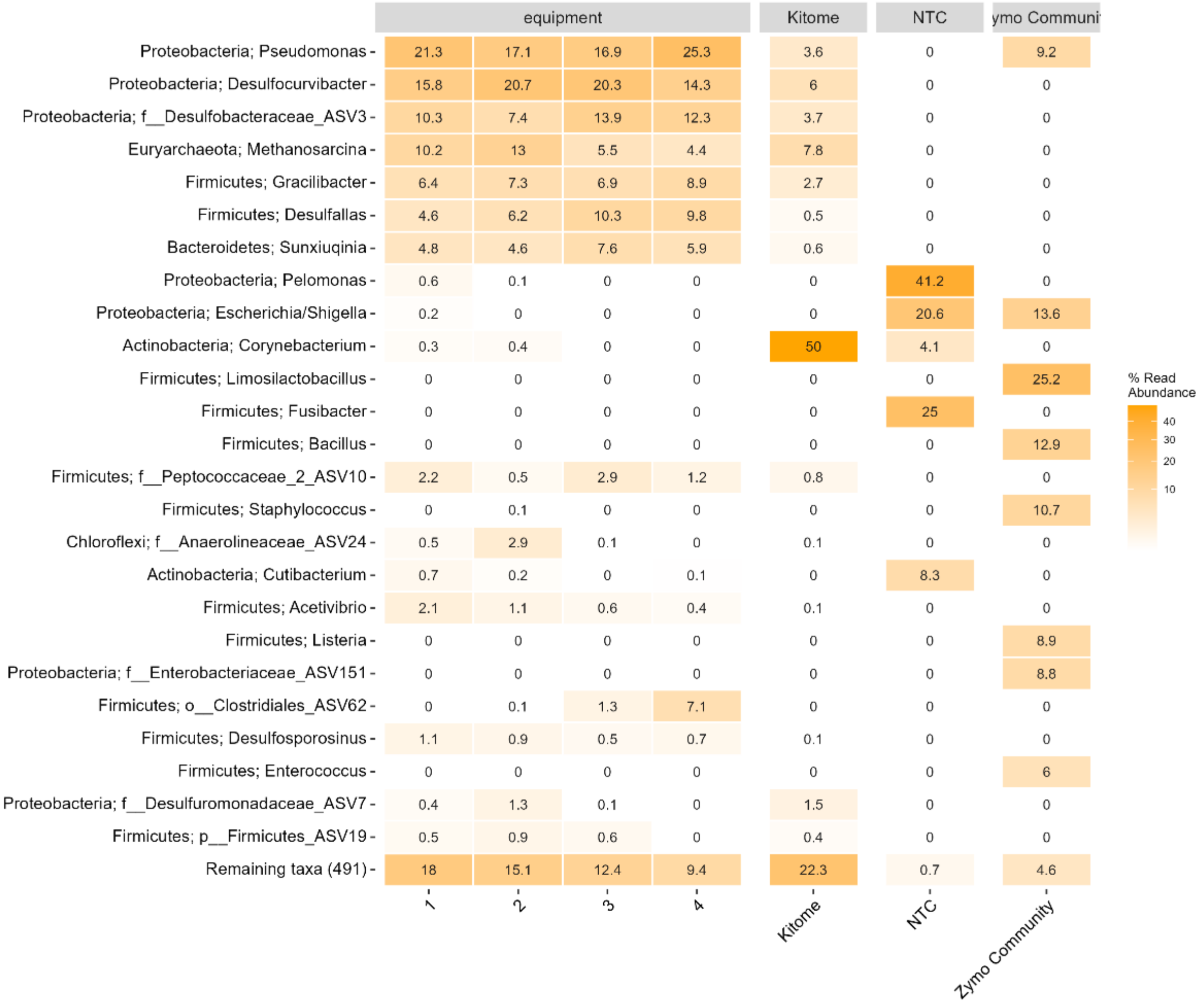

Table S13 – Statistical analysis of the radial biomass (i.e., number of 16S rRNA gene copies per gram of sand-bentonite) distribution relative to the center position of the cylindrical reactor (Excel Analysis Toolpak), C: center, OL: outer left, CL: center left, CR: center right, OR: outer right. n = 7 samples were considered for each radial position. T-test values, 0.05 are bolded.

	**Reactor 1**		**Reactor 2**		**Reactor 3**		**Reactor 4**	
	F-test	t-test	F-test	t-test	F-test	t-test	F-test	t-test
**OL vs C**	0.0003	**0.0112**	0.1332	**0.0055**	0.2384	**0.0326**	0.0010	**0.0281**
**CL vs C**	0.4090	0.1693	0.1871	0.4314	0.0525	**0.0015**	0.1196	0.1593
**CR vs C**	0.4059	0.4137	0.0982	**0.0103**	0.3854	**0.0050**	0.0107	**0.0497**
**OR vs C**	0.0550	0.0890	0.0049	**0.0391**	0.2056	0.0153	0.0000	**0.0346**

Table S14 – Heat map of ASVs relative abundances obtained from 16S rRNA gene sequencing from the initial dry materials as delivered (1 sand: 0.1-0.6mm kiln-dried quartz sand; 1 WyB: Wyoming bentonite). Results are an average of 3 replicates.

	1 sand	1 WyB
Actinobacteria; Streptomyces	0.1	27.8
Firmicutes; Bacillus	19.1	4.5
Firmicutes; Streptococcus	6.7	1.4
Firmicutes; Paraclostridium	1.2	4.4
Cyanobacteria/Chloroplast; Streptophyta	1.5	2.7
Firmicutes; Staphylococcus	1.9	2.2
Actinobacteria; Saccharopolyspora	0	4
Bacteroidetes; Prevotella	0.4	3.6
Actinobacteria; Cutibacterium	2.1	1.7
Proteobacteria; Shewanella	3.6	0
Actinobacteria; Promicromonospora	0	3.3
Actinobacteria; Corynebacterium	1.5	1.7
Proteobacteria; Pseudomonas	0.6	2.4
Firmicutes; Lactobacillus	2.9	0
Actinobacteria; Nocardioides	1.8	1
Acidobacteria; Gp6	2.4	0
Firmicutes; f__Peptostreptococcaceae_ASV246	0.3	1.8
Proteobacteria; Paracoccus	0.8	1.2
Proteobacteria; Neisseria	0.6	1.4
Actinobacteria; p__Actinobacteria_ASV195	1.9	0
Proteobacteria; Xanthomonas	0	1.8
Actinobacteria; Nocardiopsis	0	1.8
Proteobacteria; Haemophilus	1.4	0.4
Actinobacteria; Actinomadura	1.6	0
Firmicutes; f__Bacillaceae_1_ASV229	1.4	0.1
Remaining taxa (332)	46.1	30.9

% Read Abundance: 40, 30, 20, 10

References

Allan Hedin, A. H. (1997). Spent nuclear fuel - how dangerous is it?

Bagnoud, A., Chourey, K., Hettich, R. L., de Bruijn, I., Andersson, A. F., Leupin, O. X., et al. (2016). Reconstructing a hydrogen-driven microbial metabolic network in Opalinus Clay rock. *Nat Commun* 7. doi: 10.1038/ncomms12770.

Bell, E., Lamminmäki, T., Alneberg, J., Andersson, A. F., Qian, C., Xiong, W., et al. (2018). Biogeochemical Cycling by a Low-Diversity Microbial Community in Deep Groundwater. *Front. Microbiol.* 9. doi: 10.3389/fmicb.2018.02129.

Brankatschk, R., Bodenhausen, N., Zeyer, J., and Bürgmann, H. (2012). Simple Absolute Quantification Method Correcting for Quantitative PCR Efficiency Variations for Microbial Community Samples. *Appl Environ Microbiol* 78, 4481–4489. doi: 10.1128/AEM.07878-11.

Diomidis, N., Cloet, V., Leupin, O. X., Marschall, P., Poller, A., and Stein, M. (2016). Production, consumption and transport of gases in deep geological repositories according to the Swiss disposal concept. *Radioactive Waste*, 148.

Karnland, O., Olsson, S., and Nilsson, U. (2006). Mineralogy and sealing properties of various bentonites and smectite-rich clay materials.

Manca, D. (2015). Hydro-chemo-mechanical characterisation of sand/bentonite mixtures, with a focus on the water and gas transport properties. 265.

O.X. Leupin, S. S.-G. (2016). An assesment of the possible fate of gas generated in a repository for low- and intermediate-level waste.

Pearson (2002). Geochemistry of Water. 226.

Pearson, F. J., Arcos, D., Bath, A., Boisson, J.-Y., Fernández, A. M., Gaebler, H.-E., et al. (2003). *Mont Terri Project - Geochemistry of Water in the Opalinus Clay Formation at the Mont Terri Rock Laboratory*. Bern.

Ruijter, J. M., Ramakers, C., Hoogaars, W. M. H., Karlen, Y., Bakker, O., van den Hoff, M. J. B., et al. (2009). Amplification efficiency: linking baseline and bias in the analysis of quantitative PCR data. *Nucleic Acids Res* 37, e45. doi: 10.1093/nar/gkp045.
